# Induced pluripotent stem cell-derived mesenchymal stem cells activate quiescent T cells and elevate regulatory T cell response via NF-κB in allergic rhinitis patients

**DOI:** 10.1186/s13287-018-0896-z

**Published:** 2018-06-19

**Authors:** Xing-Liang Fan, Qing-Xiang Zeng, Xin Li, Cheng-Lin Li, Zhi-Bin Xu, Xue-Quan Deng, Jianbo Shi, Dong Chen, Song Guo Zheng, Qing-Ling Fu

**Affiliations:** 1 Otorhinolaryngology Hospital, The First Affiliated Hospital, Sun Yat-sen University, 58 Zhongshan Road II, Guangzhou, Guangdong 510080 People’s Republic of China; 2 Department of Emergency, Guangdong General Hospital, Guangdong Academy of Medical Science, 106 Zhongshan Road II, Guangzhou, 510080 China; 3 Department of Clinical Immunology, The Third Affiliated Hospital, Sun Yat-sen University, 600 Tianhe Road, Guangzhou, 510630 China; 4 Division of Rheumatology, Milton S. Hershey Medical Center at Penn State University, 500 University Dr. Hershey, PA 17033 USA

**Keywords:** Induced pluripotent stem cell-derived mesenchymal stem cells, Allergic rhinitis, Quiescent T cells, Immunomodulation, NF-κB

## Abstract

**Background:**

It has been demonstrated previously that induced pluripotent stem cell (iPSC)-derived mesenchymal stem cells (MSCs) have immunosuppressive effects on activated T cells. However, the effects of iPSC-MSCs on quiescent T cells are still unknown. The aim of this study was to identify the immunomodulatory role of iPSC-MSCs on resting peripheral blood mononuclear cells (PBMCs) from allergic rhinitis (AR) patients.

**Methods:**

PBMCs were cocultured with iPSC-MSCs without any stimulation, following which lymphocyte proliferation, activation of T cells, T_H_1/T_H_2 and regulatory T (Treg) cell differentiation, and Treg cell function were analyzed. The roles of soluble factors and cell–cell contact were examined to investigate the mechanisms involved.

**Results:**

iPSC-MSCs promoted the proliferation of resting lymphocytes, activated CD4^+^ and CD8^+^ T cells, and upregulated and activated Treg cells without any additional stimulation. In addition, iPSC-MSCs balanced biased T_H_1/T_H_2 cytokine levels. Cell–cell contact was confirmed to be a possible mechanism involved. NF-κB was identified to play an important role in the immunomodulatory effects of iPSC-MSCs on quiescent T cells.

**Conclusions:**

iPSC-MSCs activate quiescent T cells and elevate regulatory T-cell response in AR patients, suggesting different immunomodulatory functions of iPSC-MSCs according to the phases of diseases. Therefore, iPSC-MSCs are a potential therapeutic candidate for treating allergic airway inflammation.

**Electronic supplementary material:**

The online version of this article (10.1186/s13287-018-0896-z) contains supplementary material, which is available to authorized users.

## Background

Immunomodulatory properties of mesenchymal stromal/stem cells (MSCs) have attracted extensive attention in recent years. There have been abundant studies investigating MSC immunosuppressive function which is triggered under inflammatory conditions, i.e. interferon gamma (IFN-γ) production, which has been demonstrated to be essential for recruiting and priming MSCs [1]. Autologous, allogeneic, and even xenogeneic MSCs have shown great promise in the experimental animal models of inflammatory and immune disorder diseases [2]. Because of the absence of human leukocyte antigen (HLA)-II on cellular surfaces, allogeneic human MSCs are commonly used in clinical trials as an immunomodulator in the treatment of diseases such as organ transplantation, diabetes, multiple sclerosis, and Crohn’s disease [2]. Most experimental studies have focused on the immunosuppressive effects of MSCs on activated T cells that had been stimulated by alloantigens, mitogens, CD3/CD8 antibody, or specific antigens [3, 4]. Recently, there has been increasing interest in the MSC-mediated immunomodulatory effects on quiescent T cells. It has been reported that, in contrast to the suppressive activity on activated T cells, MSCs promoted proliferation and activation of T cells in the quiescent state [5–7].

Adult tissue-derived MSCs such as bone marrow (BM)-MSCs have limited proliferative capacities, exhibit great variability in cell quality across different donors, and lose differentiation potential very quickly [8]. All of these factors limit their therapeutic benefits [9, 10]. Recently, we successfully induced MSCs from human iPSCs (iPSC-MSCs) and identified that iPSC-MSCs exhibited lower immunogenicity, superior survival and engraftment following transplantation in a mouse ischemia model, which may be due to lower senescence compared to that of BM-MSCs [11].

Allergic rhinitis (AR) is a chronic inflammatory disorder of the nasal airways, characterized by an imbalance of T_H_1/T_H_2 cells and deficiency of regulatory T (Treg) cells [12, 13]. Adult MSC therapy is a promising candidate for the treatment of allergic airway inflammatory diseases with T_H_2-dominant responses, including AR and asthma [2]. Recently, we observed that iPSC-MSCs prevented allergic airway inflammation in a mouse model and modulated T-cell responses in an activation state in AR patients [14, 15]. However, the effects of iPSC-MSCs on immune cells under low immunogenic conditions, especially in allergic diseases, remain unclear.

The aims of the current study were to identify the immunomodulatory role of iPSC-MSCs in resting peripheral blood mononuclear cells (PBMCs) from AR patients and investigate the possible mechanisms involved in iPSC-MSC-mediated immunomodulation of resting PBMCs. In particular, the lymphocyte proliferation and T_H_1/T_H_2 and Treg cell responses from iPSC-MSC-modulated resting PBMCs were investigated.

## Methods

### Patients

This study was approved by the Ethics Committee of The First Affiliated Hospital, Sun Yat-sen University (Approval No. 2012-357) and informed consent was obtained from all participants. This study included 42 patients with AR and 39 healthy donors (normal controls). Detailed information of the patient inclusion criteria is presented in Additional file 1.

### Preparation of human iPSC-MSCs and BM-MSCs

Human iPSC-MSCs were prepared as described in our previous study [16], and the human BM-MSCs used in this study were commercially purchased from Cyagen (Guangzhou, China). Detailed information regarding human iPSC-MSC preparation is presented in Additional file 1.

### Cell culture

PBMCs were isolated from the collected heparinized peripheral blood by density gradient centrifugation using Ficoll-Paque (δ = 1.077; Amersham Biosciences, NJ, USA). In order to examine the different effects of MSCs on resting PBMCs from AR patients and healthy donors, 5 × 10^5^ isolated PBMCs were cocultured with allogeneic MSCs at different ratios in 24-well plates for 3 days without any additional mitogen or alloantigen stimulation (*n* = 12). MSCs and some of the PBMC samples utilized in this study were analyzed for HLA typing to determine the degree of mismatch. High-resolution typing for HLA-A, HLA-B, HLA-C, HLA-DRB1, and HLA-DQB1 was performed by Kindstar Global Co., Ltd (China) using PCR-sequenced based typing. Phytohemagglutinin (PHA, 5 μg/ml; Sigma, MO, USA) or phorbol 12-myristate 13-acetate (PMA, 25 ng/ml) and ionomycin (1 μg/ml; Sigma) in the coculture system were used as a positive control for lymphocyte activation where appropriate. PBMCs were collected for analysis using flow cytometry or quantitative real-time PCR (qPCR) after coculture. CellTrace™ Violet (Life Technologies, Carlsbad, CA, USA) was used to stain the cocultured MSCs, which would help to determine the purity of the collected PBMCs from the coculture system. More than 95% of the collected floating cells were PBMCs, and such density could ensure the purity of the isolated PBMCs for real-time PCR analysis. The cocultured PBMCs were harvested from the coculture systems on day 3 and cultured in a new plate for an extra 12 h. The supernatants were then harvested for cytokine concentration measurement. For MSC-secreted cytokine detection, PBMCs were removed from the coculture system on day 3, and MSCs were cultured again in the new medium for an additional 12 h. The supernatant was collected for determination of PGE2, IFN-γ, and IL-10 by ELISA.

### Lymphocyte proliferation

PBMCs were cocultured with allogeneic MSCs (either iPSC-MSCs or BM-MSCs) in 96-well plates for 3 days. PHA (5 μg/ml; Sigma) was administrated as the positive control. Effects of human MSCs on lymphocyte proliferation were examined using the thymidine incorporation assay, whereby 3H-thymidine (3H-TdR, 1 μCi (0.037 MBq); Shanghai Institute of Applied Physics, Shanghai, China) was added to the cultures 16 h prior to collection. Cells were then harvested onto glass microfiber filters and the incorporated radioactivity was measured in a 1450 Microbeta TriLux apparatus (Tri-Carb 2900RT; Perkin Elmer, Boston, MA, USA). The results were presented as the incorporated radioactivity in counts per minute (cpm). In addition, carboxyfluorescein succinimidyl ester (CFSE, 5 μM for 5 min; eBioscience, CA, USA)-stained PBMCs were cocultured with iPSC-MSCs for 3 days. The PBMCs were then harvested, stained with APC-eFluor 780-conjugated anti-CD3 (UCHT1; eBioscience), and analyzed by flow cytometry to determine the proliferation profiles. CD3^+^ T cells were first gated.

### Flow cytometry analysis

Flow cytometry was performed using a BD FACSCalibur flow cytometer (BD Biosciences, NJ, USA), in accordance with the manufacturer’s instructions, in order to identify the subtypes in lymphocytes, PBMC proliferation, and T-cell activation as well as HLA expression profiles on iPSC-MSCs. A brief description is presented in Additional file 1.

### Treg cells’ inhibitory function analysis

After 3 days of coculture with iPSC-MSCs, Treg cells were isolated from the cocultured PBMCs using the CD4^+^CD25^+^CD127^dim/−^ Regulatory T Cell Isolation Kit II (Miltenyi Biotec, Bergisch Gladbach, Germany). The isolated Treg cells were further cocultured at a ratio of 1:2 with 5 × 10^5^ allogeneic CFSE-stained PBMCs for another 3 days in the presence of PHA (5 μg/ml; Sigma) in a 24-well-plate, and then the stained PBMCs’ proliferation was determined by flow cytometry analysis, to examine the inhibitory function of the Treg cells. CD4^+^CD25^+^CD127^dim/−^ cells from PBMCs without coculture were as controls.

### Inflammatory cytokine detection

Levels of human IL-4, IL-5, IL-10, IL-13, and IFN-γ in PBMC supernatants and of IL-10, IL-13, and PGE2 in MSC supernatants were measured using enzyme-linked immunosorbent assay (ELISA) kits (R&D, MN, USA) according to the manufacturer’s instructions. The detection sensitivities were 10 pg/ml for IL-4, 0.29 pg/ml for IL-5, 3.9 pg/ml for IL-10, 32 pg/ml for IL-13, 7.8 pg/ml for IFN-γ, and 30.9 pg/ml for PGE2. Levels of IL-10 and IFN-γ in the iPSC-MSC supernatants were measured using ELISA kits from Neobioscience Technology (China). The detection sensitivities were 1 pg/ml for IL-10 and 0.8 pg/ml for IFN-γ.

### Prostaglandin inhibition

In order to determine the role of soluble factors in the stem cell immunomodulatory effects, the PGE2 inhibitor NS-398 (5 μM; Cayman Chemicals, Ann Arbor, MI, USA) was added to the coculture system to examine the role of PGE2 in MSC-mediated immunomodulation.

### Role of cell–cell contact

In order to determine the role of cell–cell contact in the immunomodulation mediated by iPSC-MSCs and BM-MSCs, MSCs (5 × 10^4^ cells/well) were plated into the lower chamber of 24-well transwell plates (Costar, Corning, NY, USA) with PBMCs (5 × 10^5^ cells/well) cultured in the upper chamber. After culturing for 3 days, cells and supernatants in the transwell were collected for flow cytometry and ELISA analysis as already described. Furthermore, vascular cell adhesion protein 1 (VCAM-1), intercellular adhesion molecule 1 (ICAM-1), and programmed cell death protein 1 (PD-1) on iPSC-MSCs were characterized so as not to leave the cell–cell contact data superficial. After coculture with PBMCs, the iPSC-MSCs were stained individually with PE-conjugated anti-VCAM-1 (STA) antibody (Biolegend, San Diego, CA, USA), FITC-conjugated anti-PD-1 (EH12.2H7) antibody (Biolegend), and PE-conjugated anti-ICAM-1 (KAT-1) antibody (eBioscience). The PBMCs were then analyzed by BD FACSCalibur flow cytometer (BD Biosciences).

### Quantitative real-time PCR

Quantitative real-time PCR (qPCR) was performed using an ABI PRISM 7500 Detection System (Applied Biosystems, Foster City, CA, USA) to determine *Foxp3*, *IL-4*, *IL-5*, and *IL-13* expression levels in PBMCs cocultured with iPSC-MSCs and BM-MSCs. A brief description is presented in Additional file 1.

### Knockdown of IKKβ in iPSC-MSCs with shRNA

IKKβ was knocked down as described in a previous report with minor modifications [17]. All procedures were done following the Biosafety Program of The First Affiliated Hospital, Sun Yat-sen University. A Biosafety Level 2+ (BSL-2+) working environment together with appropriate personal protective equipment was utilized, and caution was always taken to avoid self-inoculation during all of the related procedures. Briefly, three constructed vectors were transduced into the iPSC-MSCs. Detailed information on the constructed vectors and procedure is presented in Additional file 1.

### Statistical analysis

Statistical analysis was performed using SPSS 13.0 software for Windows (SPSS Inc., Chicago, IL, USA). One-way analysis of variance (ANOVA) followed by post hoc analysis or Dunnett T3 test for multiple comparisons with normal distribution was employed. An independent *t* test was used for comparisons between two groups. For comparisons of data with non-normal distribution, a Kruskal–Wallis rank-sum test followed by a Mann–Whitney *U* test was utilized. *P* ≤ 0.05 was considered statistically significant.

## Results

### iPSC-MSCs promoted proliferation of quiescent PBMCs

**Fig. 1:**
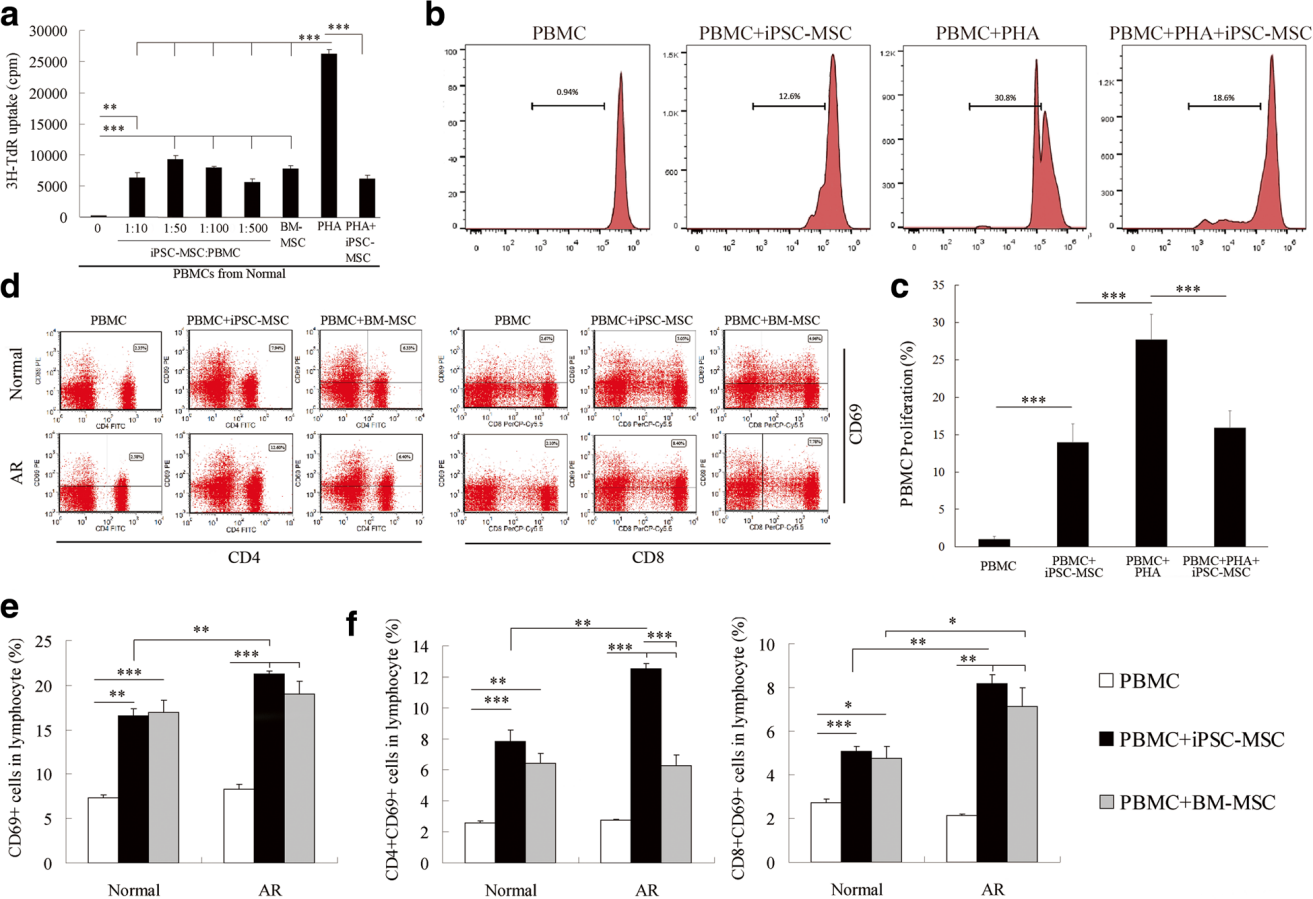
iPSC-MSCs promoted proliferation and activation of cocultured resting PBMCs from both AR patients and healthy donors. **a** iPSC-MSCs significantly promoted cocultured resting PBMC proliferation determined by thymidine incorporation assay (*n* = 6). **b** Representative flow cytometry histograms of CFSE-stained lymphocyte proliferation with treatment of iPSC-MSCs. CD3^+^ T cells first gated. **c** Statistical analysis of lymphocytes in (b) (*n* = 6). **d** Flow cytometric analysis of CD4^+^CD69^+^ and CD8^+^CD69^+^ cells in PBMCs when cocultured with different MSCs. CD3^+^ T cells first gated. **e** Statistical analysis of CD69^+^ cells in lymphocytes (*n* = 6). **f** CD4^+^CD69^+^ and CD8^+^CD69^+^ cells in lymphocytes under different culture conditions determined by flow cytometry (*n* = 6), *P < 0.05, **P < 0.01, ***P < 0.001 by Kruskal–Wallis rank-sum test followed by Mann–Whitney *U* test for two-group comparisons for (a), (c), and by one-way ANOVA and Dunnett T3 test for multiple comparisons for (e), (f). 3H-TdR 3H-thymidine, AR allergic rhinitis, BM-MSC bone marrow-derived mesenchymal stem cell, iPSC-MSC induced pluripotent stem cell-derived mesenchymal stem cell, Normal healthy donors, PBMC peripheral blood mononuclear cell, PHA phytohemagglutinin

We have demonstrated previously that iPSC-MSCs inhibited PHA-stimulated PBMC proliferation [14]. However, it is still unknown whether iPSC-MSCs could have similar inhibitory effects on quiescent T cells. In this study, the effects of iPSC-MSCs on unstimulated PBMCs were investigated. The iPSC-MSCs utilized in this study were previously demonstrated to be morphologically similar to MSCs, which showed a typical elongated fibroblast-like morphology. The iPSC-MSCs have the surface antigen profiles of MSCs (i.e., CD44^+^, CD49a^+^, CD49e^+^, CD73^+^, CD105^+^, CD166^+^, CD34^−^, CD45^−^, and CD133^−^) and display the potential for mesodermal lineage differentiations [16]. More importantly, iPSC-MSCs displayed a higher capacity for both proliferation and telomerase activity [11, 16]. When cocultured with allogeneic PBMCs from healthy subjects without any additional stimulation, iPSC-MSCs did not suppress but significantly promoted the cocultured resting PBMC proliferation at ratios of 1:10 (10^4^ MSCs vs 10^5^ PBMCs), 1:50 (2 × 10^3^ MSCs vs 10^5^ PBMCs), 1:100 (10^3^ MSCs vs 10^5^ PBMCs), and 1:500 (200 MSCs vs 10^5^ PBMCs) compared to values observed for resting PBMCs alone (Fig. 1a, *P* < 0.01 or 0.001). The ratio of 1:50 had the maximal promotion effect on resting PBMCs. Similarly, BM-MSCs (1:50) promoted the proliferation of resting PBMC proliferation (*P* < 0.001). Interestingly, these cells maintained a suppressive effect on lymphocyte proliferation after mitogen or alloantigen stimulation that was similar to those demonstrated in previous reports [14]. As shown in Fig. 1a, PHA stimulated strong lymphocyte proliferation and those iPSC-MSCs significantly inhibited lymphocyte proliferation stimulated by PHA (*P* < 0.001). It was noted that the proliferative effects induced by iPSC-MSCs and BM-MSCs were significantly weaker than those of PHA stimulation (*P* < 0.001). Proliferation rates of PBMCs treated with PHA and cocultured with MSCs as well as of the resting PBMCs treated with iPSC-MSCs were at approximately the same levels *(P >* 0.05). Similar results were drawn from the CFSE staining of PBMCs. iPSC-MSCs significantly promoted the proliferation of the cocultured resting PBMCs as determined by flow cytometry analysis. When stimulated with PHA, the PBMC proliferation was dramatically inhibited by iPSC-MSCs (Fig. 1b, c, *P* < 0.001), which was consistent with the thymidine incorporation assay. In order to determine the degree of mismatch due to the allorecognition, high-resolution typing for HLA-A, HLA-B, HLA-C, HLA-DRB1, and HLA-DQB1 of iPSC-MSCs, BM-MSCs, and some of the PBMCs utilized in this study was performed before coculture, and the HLA expression profiles on iPSC-MSCs were examined by flow cytometry both before and after coculture with the PBMCs. Additional file 1: Table S1 illustrates the HLA typing of the cells. Varying degrees of mismatches (No. ≥ 6/10) were found between PBMCs and allogeneic MSCs/PBMCs. Furthermore, according to flow cytometry analysis there was a high HLA-I expression on iPSC-MSCs, which was barely altered after coculture. Low expression of HLA-II was observed on iPSC-MSCs, and the HLA-II expression slightly increased after coculture but was still maintained at a relatively low level, which was similar to PBMCs (Additional file 1: Figure S1A, B). However, the proliferation of PBMCs cocultured with allogeneic PBMCs was less than PBMC proliferation cocultured with allogeneic iPSC-MSCs (Additional file 1: Figure S1C, D, *P* < 0.01). This at least suggests that, although the degree of mismatches was high between iPSC-MSCs and PBMCs, the effects of iPSC-MSCs to promote resting T-cell proliferation was not fully due to the allogeneic reaction. The promoting effect of iPSC-MSCs on unstimulated lymphocyte proliferation was consistent with that of adipose tissue-derived MSCs (AD-MSCs), which induced the proliferation of nonactivated or resting PBMCs [6].

### Human iPSC-MSCs activated lymphocytes from AR patients

The activation effect of iPSC-MSCs on resting PBMCs from AR patients and healthy donors was examined using flow cytometric analysis. Gated CD3^+^ T cells in PBMCs were further analyzed for expression of CD69, a T-cell early activation marker. When stimulated by PMA and ionomycin, almost 100% of the lymphocytes, CD4^+^ T cells, and CD8^+^ T cells were positive for CD69 in PBMCs isolated from both AR patients and healthy subjects (Additional file 1: Figure S2). Both human iPSC-MSCs and BM-MSCs predominantly stimulated the lymphocytes to express CD69 when cocultured with PBMCs from AR patients and healthy donors without mitogen or alloantigen stimulation (Fig. 1d and Additional file 1: Figure S3A, B). Statistical analysis demonstrated that the iPSC-MSC activation effects on lymphocytes were stronger in PBMCs of AR patients compared to the effects observed in PBMCs from healthy donors (Fig. 1e*, P* < 0.01 or 0.001). The percentages of both CD69^+^CD4^+^ T cells and CD69^+^CD8^+^ T cells were significantly increased by MSC treatment; iPSC-MSCs activated more CD4^+^ and CD8^+^ T cells, whereas BM-MSCs activated more CD8^+^ T cells in AR patients compared to those activated in healthy donors (Fig. 1f, *P <* 0.05, 0.01, or 0.001). More importantly, iPSC-MSCs exhibited stronger activation effects on CD4^+^ T cells from PBMCs of AR patients compared to the effects observed in BM-MSCs. This is probably due to the in-vitro expansion culture, which reduces the differentiation potential of adult-sourced BM-MSCs and limits their therapeutic efficacy [18]. In contrast, iPSC-MSCs are not only functional as BM-MSCs in terms of phenotype and tissue repair capability but also have been demonstrated to have similar or even stronger immunomodulation effects compared to those of adult MSCs [2], thus offering an ideal candidate for stem cell immunomodulatory therapy. The reason for MSCs exhibiting stronger promotional effects on the T cells from AR patients might be the higher numbers of allergen-specific T cells in the patients.

### iPSC-MSCs enhanced Treg cell activation in unstimulated PBMCs

**Fig. 2:**
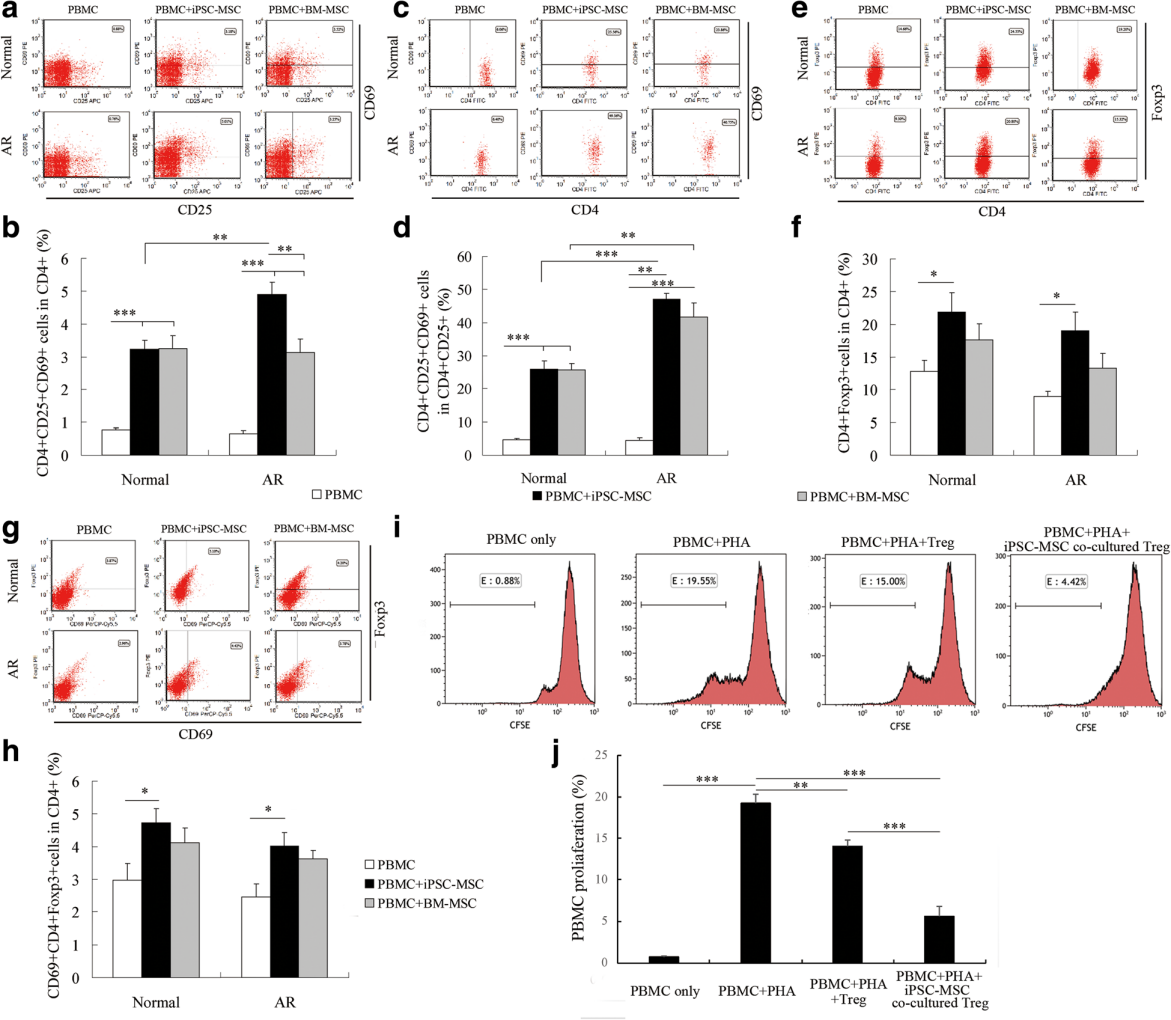
iPSC-MSCs enhanced Treg cell activation in unstimulated PBMCs. Flow cytometric analysis of CD25^+^CD69^+^ cells in CD4^+^ cells (**a**) and quantification of CD4^+^CD25^+^CD69^+^ subset percentage in CD4^+^ subpopulation (**b**) (*n* = 6). Flow cytometric analysis of CD4^+^CD69^+^ cells in CD4^+^CD25^+^ cells (**c**) and quantification of CD4^+^CD25^+^CD69^+^ subset percentages in CD4^+^CD25^+^ subpopulation (**d**) (*n* = 6). Flow cytometric analysis of CD4^+^Foxp3^+^ cells in PBMCs (**e**) and quantification of Foxp3^+^CD4^+^ Treg cells in quiescent CD4^+^ T cells (**f**) (*n* = 6). Flow cytometric analysis of CD69^+^Foxp3^+^ cells in CD4^+^ cells (**g**) and quantification of CD69^+^Foxp3^+^CD4^+^ Treg cells in quiescent CD4^+^ T cells (**h**) under different culture conditions. CD3^+^ T cells first gated (*n* = 6). **i** Representative histograms of CFSE-stained PBMC proliferation when cocultured with different conditioned Treg cells. CD3^+^ T cells first gated. **j** Statistical analysis of CFSE-stained CD3^+^ T-cell proliferation when cocultured with different conditioned Treg cells (*n* = 6). *P < 0.05, **P < 0.01, ***P < 0.001 by one-way ANOVA and Dunnett T3 test for multiple comparisons for (b), (f), (h), and by Kruskal–Wallis rank-sum test followed by Mann–Whitney *U* test for two-group comparisons for (d), (j). AR allergic rhinitis, BM-MSC bone marrow-derived mesenchymal stem cell, CFSE carboxyfluorescein succinimidyl ester, iPSC-MSC induced pluripotent stem cell-derived mesenchymal stem cell, Normal healthy donors, PBMC peripheral blood mononuclear cell, PHA phytohemagglutinin, Treg regulatory T cells

We have previously identified that human iPSC-MSCs promoted Treg cell expansion in *Dermatophagoides pteronyssinus* (Der p1)-stimulated PBMCs from AR patients who were allergic to house mite [14]. In this study, flow cytometric analysis was employed to investigate the effects of iPSC-MSCs on quiescent Treg cells without any additional stimulation. Both iPSC-MSCs and BM-MSCs significantly increased the percentage of the CD4^+^CD25^+^CD69^+^ subset in the CD4^+^ subpopulation (Fig. 2a, b and Additional file 1: Figure S3C, *P* < 0.01 or 0.001) and in the CD4^+^CD25^+^ subpopulation (Fig. 2c, d, *P* < 0.01 or 0.001) in PBMCs from both AR patients and healthy donors. Moreover, iPSC-MSC treatment exhibited stronger effects on the enhancement of CD4^+^CD25^+^CD69^+^ T cells in PBMCs from AR patients compared to that observed in normal controls *(P* < 0.01). The percentages of CD4^+^CD25^+^CD69^+^ T cells in CD4^+^ T cells of AR patients was even higher following treatment with iPSC-MSCs compared to that following treatment with BM-MSCs (Fig. 2b, *P* < 0.01), thereby demonstrating that iPSC-MSCs have a greater effect on CD4^+^CD25^+^ Treg cell activation, especially in AR patients. These data suggest that MSCs not only promote the Treg cell expansion in Der p1-stimulated PBMCs, but also enhance the proliferation of resting Treg cells.

The effects of iPSC-MSCs on Foxp3^+^CD4^+^ Treg cells were investigated. iPSC-MSC treatment significantly increased the percentage of Foxp3^+^CD4^+^ Treg cells (Fig. 2e, f and Additional file 1: Figure S4A, *P* < 0.01) and CD69^+^Foxp3^+^CD4^+^ Treg cells (Fig. 2g, h and Additional file 1: Figure S4B, *P* < 0.01) in quiescent CD4^+^ T cells from both AR patients and healthy donors *(P* < 0.05), which was consistent with the effects of iPSC-MSCs on Der p1-stimulated PBMCs in AR patients [14]. Interestingly, BM-MSC treatment did not significantly increase the Foxp3^+^CD4^+^ or CD69^+^Foxp3^+^CD4^+^ Treg cell percentages in quiescent CD4^+^ T cells. The *Foxp3* mRNA expression level in the coculture system was further investigated using quantitative real-time PCR (qPCR). There was a significantly higher level of *Foxp3* mRNA expression in unstimulated PBMCs from both AR patients and normal controls after iPSC-MSC treatment (Additional file 1: Figure S5, *P* < 0.05 or 0.01). However, no difference was observed between AR patients and normal controls with iPSC-MSC treatment in terms of *Foxp3* mRNA expression. BM-MSC treatment only increased *Foxp3* mRNA levels in PBMCs of AR patients (Additional file 1: Figure S5, *P* < 0.05), but not in healthy subjects. Human iPSC-MSCs exhibited larger effects of increasing *Foxp3* mRNA levels in healthy subjects than that observed for BM-MSCs (Additional file 1: Figure S5, *P* < 0.05).

We further validated the inhibitory function of the Treg cells cocultured with iPSC-MSCs on T-cell proliferation using CFSE staining. Treg cells isolated from PBMCs without coculture with iPSC-MSCs showed some inhibitory function to CD3^+^ T-cell proliferation. However, the Treg cells cocultured with iPSC-MSCs exhibited more inhibition on the CD3^+^ T-cell proliferation compared to those in the PHA group and Treg cells without coculture with iPSC-MSCs (Fig. 2i, j, *P* < 0.01 and 0.001). This suggests that the upregulated Treg cells treated with iPSC-MSCs have the inhibitory function.

Previous studies showed that Treg cells are immunosuppressive and generally suppress or downregulate induction and proliferation of effector T cells [19]. MSC coculture promoted the proliferation of resting CD4^+^/CD8^+^ T cells and Treg cells at the same time, which maintained the ratio of allergen-specific T_H_2 cells and allergen-specific Treg cells, thus maintaining the development of a healthy immune response.

### iPSC-MSCs regulated cytokine production in unstimulated PBMCs

**Fig. 3:**
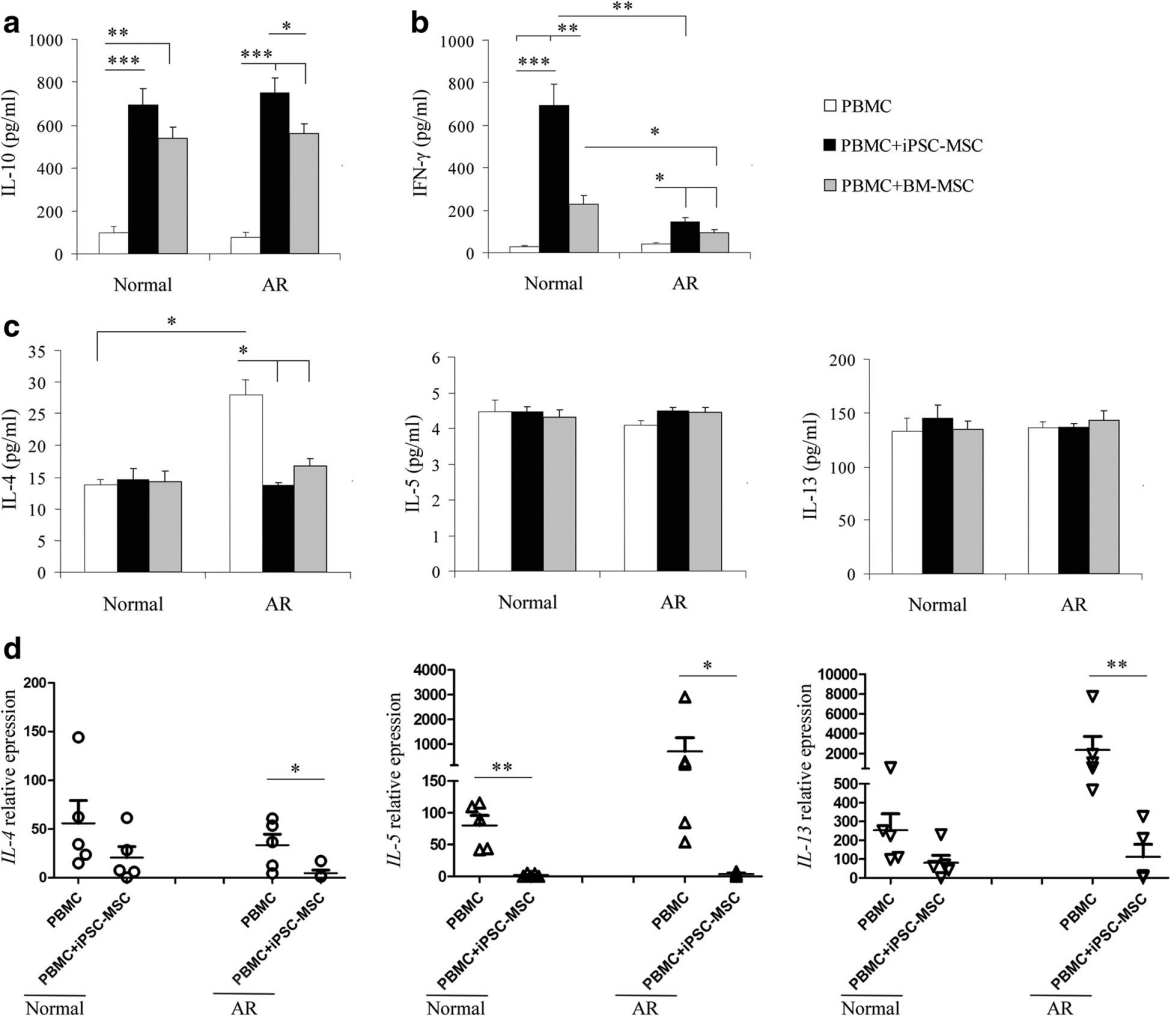
iPSC-MSCs regulated cytokine production in unstimulated PBMCs. **a** IL-10 secretion levels in PBMCs under different culture conditions determined by ELISA (*n* = 6). **b** IFN-γ secretion levels in PBMCs under different culture conditions determined by ELISA (*n* = 6). **c** T_H_2-type cytokine (IL-4, IL-5, and IL-13) secretion levels in PBMCs under different culture conditions determined by ELISA (*n* = 6). **d** *IL-4*, *IL-5*, and *IL-13* mRNA expression levels were decreased after coculture with iPSC-MSCs determined by qPCR (*n* = 6). *P < 0.05, **P < 0.01, ***P < 0.001, by Kruskal–Wallis rank-sum test followed by Mann–Whitney *U* test for two-group comparisons for (a), (b), by one-way ANOVA and Dunnett T3 test for multiple comparisons for (c), and by independent *t* test for (d). AR allergic rhinitis, BM-MSC bone marrow-derived mesenchymal stem cell, IFN-γ interferon gamma, IL interleukin, iPSC-MSC induced pluripotent stem cell-derived mesenchymal stem cell, Normal healthy donors, PBMC peripheral blood mononuclear cell

We have demonstrated that iPSC-MSCs upregulated the levels of IL-10 and IFN-γ (representing the T_H_1 phenotype) and downregulated IL-4, IL-5, and IL-13 (representing the T_H_2 phenotype) when cocultured with Der p1-stimulated PBMCs from AR patients [14]. In this study, the cocultured PBMCs were harvested from the coculture systems on day 3 and cultured in a new plate for an extra 12 h to examine the cytokine expression profiles of PBMCs. The PBMCs collected from AR patients and healthy donors maintained basal levels of IL-10, IFN-γ, IL-4, and IL-5 without any stimulation. iPSC-MSCs or BM-MSCs significantly increased IL-10 levels in PBMCs from both AR patients and healthy donors (Fig. 3a, *P* < 0.05, 0.01, or 0.001), which showed good agreement with the increased Treg cell percentage according to flow cytometric analysis. IL-10 levels from AR patients were higher in the iPSC-MSC group compared to those in the BM-MSC group (*P* < 0.05). IFN-γ levels were also increased in PBMCs from AR patients and healthy donors with MSC coculture (Fig. 3b, *P* < 0.05, 0.01, or 0.001), and higher levels of IFN-γ were detected in PBMCs from healthy donors than those from AR patients. Furthermore, MSC treatment significantly decreased IL-4 levels in PBMCs from AR patients (Fig. 3c, *P* < 0.05), but not in those from healthy donors. IL-5 and IL-13 levels were not influenced by MSC treatment either in PBMCs from AR patients or in those from healthy donors (Fig. 3c). Because MSCs are also able to secrete IL-10 and IFN-γ, the levels of IL-10 and IFN-γ secreted by the cocultured iPSC-MSCs were also determined. PBMCs were removed from the coculture systems on day 3, and iPSC-MSCs were cultured again in the new medium for an additional 12 h. A very small amount of IL-10 (Additional file 1: Figure S6A) and no detectable IFN-γ (data not shown) was identified. Furthermore, quantitative real-time PCR was carried out to determine *IL-4*, *IL-5*, and *IL-13* expression levels in PBMCs cocultured with iPSC-MSCs. *IL-4*, *IL-5*, and *IL-13* levels were also confirmed to be decreased at the genetic level after coculture (Fig. 3d, *P* < 0.05). Of course, the effect of allorecognition involved should be evaluated. We therefore cocultured PBMCs with allogeneic PBMCs and found that the levels of IL-10 and IFN-γ in the supernatant had no change after coculture (Additional file 1: Figure S6B). This suggests that alloreactivity was not a major aspect to affect the production of IL-10 and IFN-γ of PBMCs cocultured with allogeneic iPSC-MSCs. These results indicate that iPSC-MSCs not only promote the proliferation and activation of unstimulated T_H_1 and T_H_2 cells but also regulate T_H_2 cytokine secretion simultaneously. The immunomodulatory effects of MSCs promote effector T-cell proliferation in resting PBMCs, and regulate their functions by enhancing Treg cells and T_H_1 cell function. These actions may help keep the patients’ immune response in a healthy status.

### The role of cell–cell contact and soluble factors in iPSC-MSC-mediated immunomodulation

**Fig. 4:**
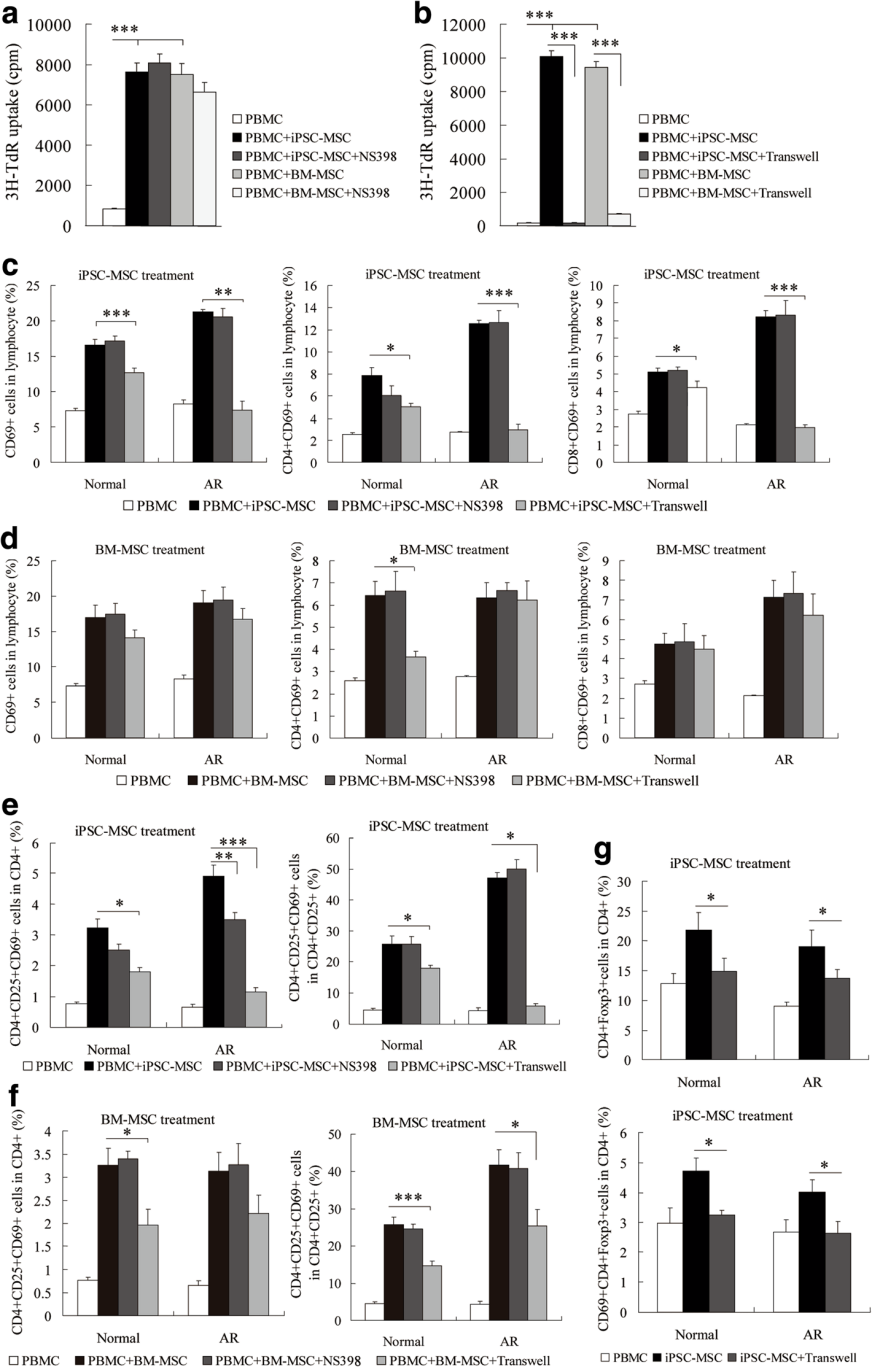
Role of PGE2 and cell–cell contact in iPSC-MSC-mediated immunomodulation. PBMC proliferation when cocultured with iPSC-MSCs and NS398 (**a**) or with iPSC-MSCs in transwell (**b**) determined by thymidine incorporation assay. Statistical analysis of activated T cells in lymphocytes when cocultured with iPSC-MSCs (**c**) or BM-MSCs (**d**) under different conditions determined by flow cytometry. **e** Statistical analysis of activated Treg cells in CD4^+^ cells when cocultured with iPSC-MSCs (**e**) or BM-MSCs (**f**) under different conditions determined by flow cytometry. **g** Statistical analysis of CD69^+^CD4^+^Foxp3^+^ cells in CD4^+^ cells when cocultured with iPSC-MSCs in transwell determined by flow cytometry. CD3^+^ T cells first gated. (*n* = 6 for each statistical analysis). *P < 0.05, **P < 0.01, ***P < 0.001 by Kruskal–Wallis rank-sum test followed by Mann–Whitney *U* test for two-group comparisons for (a), (b), and by one-way ANOVA and Dunnett T3 test for multiple comparisons for (c)–(g). 3H-TdR 3H-thymidine, AR allergic rhinitis, BM-MSC bone marrow-derived mesenchymal stem cell, cpm counts per minute, iPSC-MSC induced pluripotent stem cell-derived mesenchymal stem cell, Normal healthy donors, NS398 COX-2 inhibitor, PBMC peripheral blood mononuclear cell

We previously identified that cell–cell contact and prostaglandin E2 (PGE2) were associated with the iPSC-MSC-mediated immunomodulatory properties on Der p1-stimulated PBMCs from AR patients [14]. In this study, we continued focusing on the mechanisms of cell–cell contact and soluble factors that are involved in iPSC-MSC-mediated immunomodulation of resting PBMCs. We observed that PGE2 levels were dramatically increased in unstimulated PBMCs from both AR patients and healthy donors when cocultured with MSCs, while PGE2 levels were significantly decreased following the addition of a specific COX-2 inhibitor, namely NS398, in the coculture system (Additional file 1: Figure S7). Therefore, NS398 was able to block the iPSC-MSC-mediated immunomodulatory effect. However, the addition of NS-398 failed to prevent iPSC-MSCs or BM-MSCs from exerting enhanced effects on resting lymphocyte proliferation (Fig. 4a). When cocultured in 24-well transwells, there was a greater difference in lymphocyte proliferation between the mixed cells with cell–cell contact group and the separated cells without cell–cell contact group; lymphocyte proliferation in PBMCs was dramatically reduced without cell–cell contact (Fig. 4b, *P* < 0.001), indicating that cell–cell contact was required for resting lymphocyte proliferation that was initiated by MSCs.

The role of NS-398 and cell–cell contact in MSC-mediated immunomodulation effects on resting lymphocyte subsets such as CD3^+^ T cells, CD4^+^ T cells, CD8^+^ T cells, and CD4^+^CD25^+^ and Foxp3^+^CD4^+^ Treg cells was then investigated using flow cytometric analysis. When separated in the transwells, iPSC-MSC-mediated activation effects on lymphocytes, CD4^+^ T cells, CD8^+^ T cells, and Treg cells were significantly blocked in both quiescent PBMCs from AR patients and healthy donors (Fig. 4c, e, *P* < 0.05, 0.01, or 0.001). However, transwell coculture only blocked BM-MSC-mediated activation effects on CD4^+^CD69^+^ T cells in lymphocytes (Fig. 4d, *P* < 0.05) and CD69^+^CD4^+^CD25^+^ in CD4^+^ T cells from healthy donors, and CD69^+^CD4^+^CD25^+^ in CD4^+^CD25^+^ T cells from AR patients and healthy donors (Fig. 4f, *P* < 0.05, 0.001). Moreover, the percentages of CD4^+^Foxp3^+^ and CD69^+^CD4^+^Foxp3^+^ in CD4^+^ T cells were significantly decreased after separating iPSC-MSCs from PBMCs (Fig. 4g, all *P* < 0.05). Furthermore, VCAM-1, ICAM-1, and PD-1 on iPSC-MSCs were characterized by flow cytometry analysis after coculture with PBMCs. VCAM-1 and PD-1 expression on iPSC-MSCs was slightly increased after coculture with PBMCs, and the addition of NS398 further increased the expression of VCAM-1 and PD-1 on iPSC-MSCs compared to that of iPSC-MSC control. However, no significant difference of the VCAM-1, ICAM-1, and PD-1 expression was found between the iPSC-MSCs and PBMC-cocultured iPSC-MSCs (Additional file 1: Figure S8). Although MSC-derived PGE2 played a role in Treg cell proliferation, as reported previously [1, 20]. The current study found that NS398 only blocked the iPSC-MSC-mediated enhanced effects on CD4^+^CD25^+^CD69^+^ T cells from AR patients (Fig. 4e, *P* < 0.01). These data demonstrate that cell–cell contact is essential for iPSC-mediated immunomodulation in quiescent PBMCs, which is required for the MSC-enhanced proliferation of CD4^+^ T cells, CD8^+^ T cells, and Treg cells.

### NF-κB was critical for iPSC-MSC-mediated immunomodulation

**Fig. 5:**
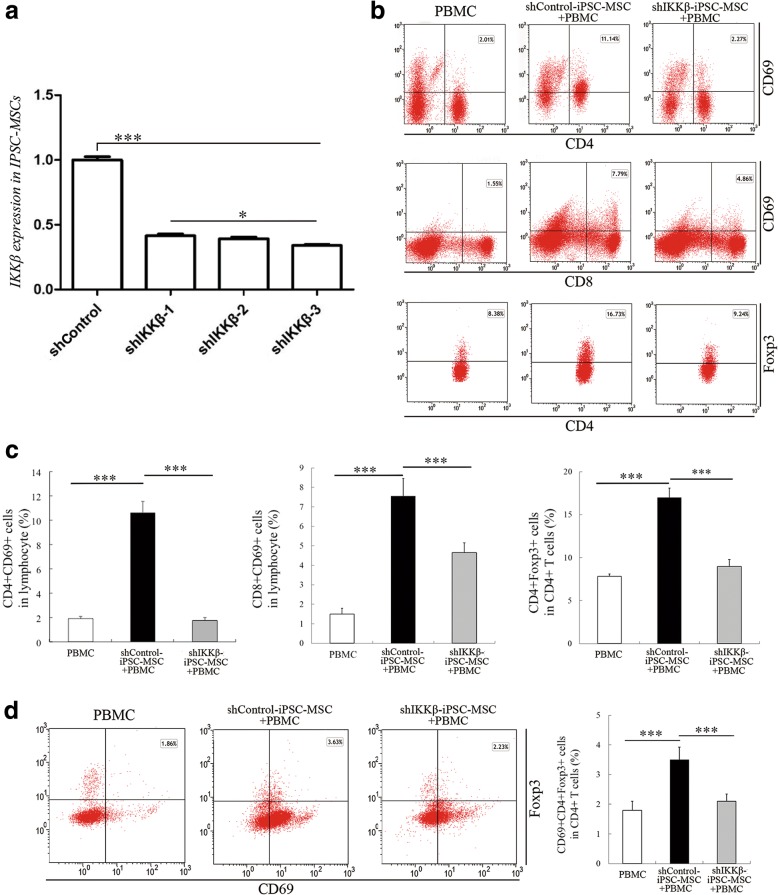
Role of NF-κB in iPSC-MSC-mediated immunomodulation. PBMCs from AR patients cocultured with shIKKβ-iPSC-MSCs. **a** qPCR analysis of *IKKβ* expression in iPSC-MSCs after lentiviral transduction (*n* = 6). **b** Flow cytometric analysis of activated CD4^+^ and CD8^+^ T cells in lymphocytes and Treg cells in CD4^+^ T cells with CD69^+^ cells when cocultured with shIKKβ-iPSC-MSCs. **c** Statistical analysis of activated CD4^+^ and CD8^+^ T cells in lymphocytes and Treg cells in CD4^+^ T cells when cocultured with shIKKβ-iPSC-MSCs (*n* = 6). **d** Flow cytometric and statistical analysis of CD69^+^Foxp3^+^CD4^+^ Treg cells in CD4^+^ T cells when cocultured with shIKKβ-iPSC-MSCs. CD3^+^ T cells first gated (*n* = 6). *P < 0.05, ***P < 0.001 by one-way ANOVA and Dunnett T3 test for multiple comparisons. iPSC-MSC induced pluripotent stem cell-derived mesenchymal stem cell, PBMC peripheral blood mononuclear cell, sh short hairpin

NF-κB plays a critical role in cell survival, proliferation, inflammation, and immunity [21]. It is recognized that the NF-κB signaling pathway was involved in BM-MSC-mediated immunosuppressive effects [22]. However, the role of NF-κB in iPSC-MSC-mediated immunomodulation of T cells including quiescent T cells is still unknown. Therefore, iPSC-MSCs transduced with a vector containing short hairpin RNA (shRNA) for IKKβ (shIKKβ-iPSC-MSCs) were cocultured with the resting PBMCs from AR patients. IKKβ was silenced because the IKK kinase complex is the core element of the NF-κB cascade [23]. After stable transduction, qPCR was used to examine the knockdown of IKKβ. All constructed shIKKβ vectors were effective at silencing IKKβ expression in iPSC-MSCs, and shIKKβ-3 demonstrated the highest efficiency (Fig. 5a, *P* < 0.05 or 0.001). Therefore, shIKKβ-3-transduced iPSC-MSCs were further cocultured with PBMCs from AR patients to investigate the role of NF-κB in iPSC-MSCs. The shControl-iPSC-MSCs significantly increased the percentages of CD4^+^CD69^+^ and CD8^+^CD69^+^ T cells in lymphocytes; the percentages of CD4^+^Foxp3^+^ cells, as well as the CD69^+^CD4^+^Foxp3^+^ cells, in CD4^+^ T cells were also increased compared with those in PBMCs only. However, reduction of IKKβ in iPSC-MSCs significantly reversed the cell activation effects on resting PBMCs with decreased levels of CD4^+^CD69^+^ and CD8^+^CD69^+^ cell percentages in lymphocytes (Fig. 5b, c, both *P* < 0.001). More importantly, CD4^+^Foxp3^+^ and CD69^+^CD4^+^Foxp3^+^ cells in the CD4^+^ T cells were also significantly reversed, which was almost similar to those in only PBMCs (Fig. 5b–d, *P* < 0.001 compared to shControl-iPSC-MSCs). These findings suggest that the NF-κB signaling pathway is critical for iPSC-MSC-mediated immunomodulation on quiescent T cells including Treg cells.

## Discussion

The immunosuppressive effects of MSCs (primarily BM-MSCs) on mitogen-activated immune cells have been widely studied [24–26]. However, a previous study found that MSCs either inhibited lymphocyte growth when the initial stimulus is high enough, or promoted lymphocyte proliferation when the initial stimulus is absent or low [27]. Only a few studies have focused on the effects of MSCs on immune cells under low immunogenic conditions, and they demonstrated that MSCs induced proliferation and promoted survival of unstimulated T cells [5, 6]. We previously reported that iPSC-MSCs inhibited proliferation of PBMCs under the activation of mitogen [14]. However, it is important to know the full range of the immunomodulatory effects exerted by iPSC-MSCs for clinical applications. This study demonstrated that resting PBMC proliferation was increased by coculture with allogeneic iPSC-MSCs but was much lower than the effect observed in mitogen-stimulated PBMCs. This suggests that MSCs promote proliferation of resting PBMCs but at a relatively low level. Both iPSC-MSCs and BM-MSCs were demonstrated to activate unstimulated lymphocytes, CD4^+^ T cells, and CD8^+^ T cells from healthy donors when using CD69 as the activation marker. CD69 is rapidly expressed upon T-cell activation and is readily amenable to detection by flow cytometry, suggesting its utility as a marker for the rapid assessment of T-cell activation [28]. Moreover, both types of MSCs increased the activation of resting CD4^+^CD25^+^ T cells, and iPSC-MSCs increased the percentage of CD4^+^Foxp3^+^ Treg cells and promoted their activation. The results are consistent with the previous study which demonstrated that AD-MSCs stimulated resting T cells and enhanced Treg cell generation [6]. Taken together, these data indicate that MSCs might exert different effects, different strengths, and even opposing effects depending on the state of T cells, thus achieving a therapeutic function in different clinical scenarios.

Nevertheless, previous studies only focused on the effects of MSCs on resting T cells from normal subjects. This study investigated the effects of iPSC-MSCs on unstimulated T cells from both healthy volunteers and AR patients. It was identified that iPSC-MSCs had stronger activation effects on resting lymphocytes, CD4^+^ T cells, and CD4^+^CD25^+^ T cells in PBMCs from AR patients compared to those from healthy controls. To the best of our knowledge, this was the first study to investigate the effects of MSCs on resting T cells from patients with allergic diseases. Furthermore, the results from flow cytometry and qPCR analyses demonstrated that iPSC-MSCs increased the percentage of CD4^+^Foxp3^+^ Treg cells and promoted their activation. We have reported that allogeneic iPSC-MSCs were able to modulate allergen-stimulated T-cell phenotypes from AR patients toward T_H_2 cell suppression by inducing Treg cell expansion [14]. Here, we confirmed that even without any stimulation, iPSC-MSCs were able to generate Treg cells, improve T_H_1 cell responsiveness, decrease T_H_2 cytokine (IL-4) secretion, and decrease mRNA levels of IL-4, IL-5, and IL-13.

In our study, we cocultured PBMCs with allogeneic MSCs. HLA-typing experiments showed that varying degrees of mismatches were found between PBMCs and iPSC-MSCs/BM-MSCs/allogeneic PBMCs. The alloreactivity from the mismatch of PBMCs and allogeneic MSCs may contribute to the effects of MSCs to stimulate resting T cells. We reported previously that iPSC-MSCs expressed low expression of HLA-II even with the stimulation of IFN-γ [11]. In this study, we identified that there was low expression of HLA-II on iPSC-MSCs that was slightly increased after coculture with PBMCs, but still maintained at a relatively low level which was similar to PBMCs. We further identified that the proliferation rate of PBMCs cocultured with allogeneic PBMCs was lower than the PBMCs cocultured with allogeneic iPSC-MSCs. Additionally, we found that PBMCs cocultured with allogeneic iPSC-MSCs produced more IFN-γ and IL-10. However, there was no change for IFN-γ and IL-10 after PBMCs were cocultured with allogeneic PBMCs. Furthermore, we identified that the Treg cells cocultured with allogeneic iPSC-MSCs had strong inhibition effects on T-cell proliferation. Our data at least suggest that under a high degree of mismatches between iPSC-MSCs and PBMCs, the activity of iPSC-MSCs to promote resting T cells was not only because of the allogenicity. Alternatively, allogenicity may have activity, but with no more effects for PBMCs when cocultured with allogeneic MSCs.

The absence or presence of allergens mimics the progress of patients with AR in a quiescent state or under exposure to allergens, respectively. Traditionally, AR patients have been classified into seasonal and perennial categories. However, minimal persistent inflammation (MPI) appears to be present year-round in seasonal and perennial AR patients with inflammatory cell infiltration in the nasal mucosa but without allergic symptoms [29]. MPI can be considered a persistent state of AR patients with subclinical inflammation without exposure to allergens or with subthreshold doses of allergen stimulation [30]. Moreover, MPI may also contribute to hyperactivity, increased susceptibility to development of clinical symptoms, as well as comorbidities of AR (i.e., asthma) [31]. Additionally, in light of the clear relationship between the upper and lower airways, the relevance of nasal MPI to lower airway inflammation must be considered [32]. Therefore, more attention should be paid to inhibit MPI to prevent the induction or exacerbation of clinical AR. The culture system used in this study examined T cells without allergen stimulation, in part reflecting the state of MPI in AR, and also revealing the potential immunomodulatory effects of iPSC-MSCs in the clinical setting.

It was demonstrated that CD4^+^CD25^+^Foxp3^+^ natural Treg cells and inducible type 1 Treg cells inhibit the development of allergy via several pathways [33, 34]. CD4^+^CD25^+^ Treg cells were observed to inhibit allergen-specific responses to the house dust mite allergen in the mouse model of air inflammation [35]. Existing therapies for allergic diseases, such as treatment with glucocorticoids and allergen-specific immunotherapy, are associated with the induction of CD4^+^CD25^+^ Treg cells [36]. Here, we observed that human iPSC-MSCs upregulated the percentage of CD4^+^CD25^+^ Treg cells in resting PBMCs from both AR patients and healthy donors, which was in line with previous studies demonstrating that adult MSCs induced an increase in Treg cells [37]. More importantly, we identified that the Treg cells cocultured with the iPSC-MSCs exhibited a significant inhibitory function on T-cell proliferation, which suggests the strong effects of iPSC-MSCs to promote the successful inhibitory functions of Treg cells in the quiescent situation. As we reported previously [38], in the coculture of mouse T cells and human iPSC-MSCs without any stimuli, the proliferation of T cells was promoted; however, the percentages of T_H_1 and T_H_2 cells and the primed cytokine expression were decreased. Meanwhile, the T_H_17 and Treg cell frequencies were increased, suggesting that iPSC-MSCs may inhibit allergic inflammation via upregulation of Treg cells. Similar behavior was also observed for adult MSCs in the mouse AR model [39]. In our study, the upregulation effect of iPSC-MSCs on the frequency of Treg cells in the resting condition may contribute to the T_H_2 immune response inhibition. We indeed observed that iPSC-MSCs decreased T_H_2 cytokine production despite the activation of T cells. This suggests that upregulation of functional Treg cells in the activation of T cells may play a dominant role and then gradually downregulate the T_H_2 immune response in AR patients. These data indicate that either for quiescent or activated states, iPSC-MSCs prevent the T_H_2 immune response for AR patients. Current evidence suggests that allogeneic human MSCs are able to exert adaptable immunomodulatory effects on the same types of immune cells depending on the local microenvironment or disease status. For instance, the T_H_1 cell response in patients with acute graft versus host disease (GvHD) was significantly decreased by MSCs [40]. However, both BM-MSCs and iPSC-MSCs lead to a shift from the T_H_2 to the T_H_1 cell response in airway allergic inflammatory diseases [14, 15]. These observations suggest that MSCs are able to “sense” their immunological environment and switch their effects to protect the body from the disease in different situations.

The primary mechanisms by which MSCs modulate T-cell responses have been suggested to include cell–cell contact and soluble factors. Cell–cell contact may be required for maximum T-cell suppression by MSCs; however, soluble factors secreted by MSCs have recently been demonstrated to play a key role in MSC-meditated immune suppression [41–43]. A previous report demonstrated that a PGE2 inhibitor, but not a TGF-β neutralizing antibody, was able to reverse the inhibitory effect of adult MSCs on lymphocyte proliferation [44]. Similar to adult MSCs, several other soluble factors including hepatocyte growth factor, indoleamine 2,3-dioxygenase, nitric oxide, and IL-10 might also mediate the immunosuppressive effect of iPSC-MSCs [1, 45]. In this study, we observed that cell–cell contact has played an important role in iPSC-mediated immunomodulation in quiescent PBMCs, although soluble factor PGE2 somehow also exerts its action.

Allogeneic human MSCs are able to adjust their immunomodulatory behaviors according to the different local microenvironment or disease status, so they may exert different effects even on the same types of the immune cells [2]. This raises a question regarding the mechanisms underlying the active immunomodulatory effects of iPSC-MSCs in this study on resting lymphocytes and T cells. MSC-mediated immunomodulation is complex, and multiple regulatory mechanisms exist without an obvious hierarchy of importance [22]. NF-κB played a pivotal role in a variety of biological processes including innate and adaptive immunity [46]. NF-κB regulates multiple aspects of innate and adaptive immune functions and serves as a pivotal mediator of inflammatory responses in immune cells [47]. Researchers have demonstrated that the NF-κB signaling pathway in BM-MSCs was involved in BM-MSC-mediated immunosuppressive effects [22, 48]. However, the role of NF-κB in iPSC-MSC-mediated immunomodulation in resting T cells is still unknown. Therefore, this study investigated the function of NF-κB in association with immunomodulatory effects of MSCs, and highlighted the iPSC-MSC-mediated immunomodulation on resting T cells. In order to investigate the role of NF-κB in iPSC-MSCs, three specific shRNA lentivirus vectors targeting IKKβ were established, and iPSC-MSCs were transduced with the IKKβ-targeted lentivirus to inhibit the NF-κB signaling pathway in cells. When IKKβ was silenced in iPSC-MSCs, the upregulation effects on activated CD4^+^ and CD8^+^ cells, especially for Treg cells, were dramatically inhibited. These results indicate that the NF-κB signaling pathway in iPSC-MSCs promoted resting T-cell proliferation and activation. Therefore, the NF-κB signaling pathway in MSCs is not only important for MSC-mediated immunosuppressive effects, but also for iPSC-MSC-mediated resting T-cell activation. Moreover, iPSC-MSC-based inflammatory disease-targeting strategies may be adapted for different T-cell states.

## Conclusion

The immunomodulatory effects of iPSC-MSCs on resting PBMCs in terms of lymphocyte proliferation and T_H_1/T_H_2 and Treg cell responses from AR patients were identified in this study. In contrast to the inhibition of immune responses observed in activated PBMCs, iPSC-MSCs promoted the responses of lymphocytes especially for Treg cells and eventually balanced the biased T_H_1/T_H_2 immunity in quiescent-state PBMCs from AR patients by direct cell–cell contact. The NF-κB signaling pathway was also observed to be critical for iPSC-MSC-mediated immunomodulation in resting T cells. This suggests that iPSC-MSCs are capable of exerting different immunomodulatory effects according to the phases of diseases, which further illustrates the therapeutic potential of MSCs for treating allergic airway inflammation.

## Additional file

Additional file 1: Additional methods. **Table S1.** High-resolution HLA typing for HLA-A, HLA-B, HLA-C, HLA-DRB1, and HLA-DQB1 of MSCs and PBMCs utilized in this study. **Figure S1.** Promotion effects of iPSC-MSCs on resting PBMCs were not only due to allogeneic recognition. **Figure S2.** Activation of CD4^+^ and CD8^+^ T cells in both AR patients’ and normal PBMCs with stimulation of PMA and ionomycin. **Figure S3.** Representative gating strategies for CD4^+^ and CD8^+^ T cells in this study. **Figure S4.** Representative gating strategies for Treg cells in this study. **Figure S5.** *Foxp3* mRNA expression in PBMCs from normal and AR patients after coculture with MSCs. **Figure S6.** IL-10 and IFN-γ levels secreted by iPSC-MSCs/PBMCs under different conditions. **Figure S7.** PGE2 levels in PBMCs from healthy control and AR patients when cocultured with MSCs and NS398. **Figure S8.** ICAM-1, VCAM-1, and PD-1 expression on iPSC-MSCs when cocultured with PBMCs (DOCX 14612 kb)

## Abbreviations

3H-TdR: 3H-thymidine
AR: Allergic rhinitis
BM: Bone marrow
ELISA: Enzyme-linked immunosorbent assay
GvHD: Graft versus host disease
HLA: Human leukocyte antigen
ICAM-1: Intercellular adhesion molecule 1
IFN-γ: Interferon gamma
iPSC: Induced pluripotent stem cell
MSC: Mesenchymal stem cell
PBMC: Peripheral blood mononuclear cell
PD-1: Programmed cell death protein 1
PGE2: Prostaglandin E2
PHA: Phytohemagglutinin
PMA: Phorbol 12-myristate 13-acetate
qPCR: Quantitative real-time PCR
Treg: Regulatory T
VCAM-1: Vascular cell adhesion protein 1
