# Supplementary material for: Induced pluripotent stem cell-derived mesenchymal stem cells activate quiescent T cells and elevate regulatory T cell response via NF-κB in allergic rhinitis patients
Source: Stem Cell Res Ther. 2018 Jun 19;9:170. doi: 10.1186/s13287-018-0896-z (PMC6010028; doi:10.1186/s13287-018-0896-z)
Supplement: Supplementary file 1 — Additional methods. Table S1. High-resolution HLA typing for HLA-A, HLA-B, HLA-C, HLA-DRB1, and HLA-DQB1 of MSCs and PBMCs utilized in this study. Figure S1. Promotion effects of iPSC-MSCs on resting PBMCs were not only due to allogeneic recognition. Figure S2. Activation of CD4+ and CD8+ T cells in both AR patients’ and normal PBMCs with stimulation of PMA and ionomycin. Figure S3. Representative gating strategies for CD4+ and CD8+ T cells in this study. Figure S4. Representative gating strategies for Treg cells in this study. Figure S5. Foxp3 mRNA expression in PBMCs from normal and AR patients after coculture with MSCs. Figure S6. IL-10 and IFN-γ levels secreted by iPSC-MSCs/PBMCs under different conditions. Figure S7. PGE2 levels in PBMCs from healthy control and AR patients when cocultured with MSCs and NS398. Figure S8. ICAM-1, VCAM-1, and PD-1 expression on iPSC-MSCs when cocultured with PBMCs (DOCX 14612 kb) [file 13287_2018_896_MOESM1_ESM.docx]

Additional file 1

Induced pluripotent stem cell-derived mesenchymal stem cells activate quiescent T cells and elevate regulatory T cell response via NF-κB in allergic rhinitis patients

Xing-Liang Fan, Qing-Xiang Zeng, Xin Li, Cheng-Lin Li, Zhi-Bin Xu, Xue-Quan Deng, Jianbo Shi, Dong Chen, Song Guo Zheng^*^, Qing-Ling Fu^*^

*** Correspondence:** Song Guo Zheng: [szheng1@hmc.psu.edu](mailto:szheng1@hmc.psu.edu) & Qing-Ling Fu: [fuqingl@mail.sysu.edu.cn](mailto:fuqingl@mail.sysu.edu.cn)

# Additional methods

**Patient inclusion criterion**

The diagnosis of allergic rhinitis (AR) was in accordance with the criteria of the initiative from Allergic Rhinitis and its Impact on Asthma (ARIA) (Bousquet et al., 2008). Atopy was defined both positive for skin prick test (SPT) result to *Dermatophagoides pteronyssinus* (Der p1) and positive for specific immunoglobulin E (IgE) to Der p1 (Pharmacia CAP System, Pharmacia Diagnostics, Uppsala, Sweden). The patients should have neither had received antihistamines or intranasal steroid treatments for at least 1 week, nor oral steroids for three months prior to the study. Healthy controls should have had none of any symptoms, positive SPT or specific IgE to Der p1.

**Preparation of human iPSC-MSCs and BM-MSCs**

Human urine cell-derived iPSCs were generated by electroporating plasmid pEP4EO2SET2K which contained OCT4, SOX2, SV40LT and KLF4 into the cells (Xue et al., 2013). The generated iPS colonies were kept in mTeSR1 (Stem Cell Technologies, Vancouver, Canada) supplemented with 50 U/mL of penicillin G and 50 mg/mL of streptomycin (Gibco, Invitrogen Corporation, Carlsbad, CA) until 60% confluency, the medium was then replaced by MSC-inducing medium that contained 90% Minimum Essential Medium Eagle-α modified (α-MEM, Gibco), 10% serum replacement (Stem Cell Technologies, Vancouver, Canada), 1% penicillin/streptomycin, 1 mM sodium pyruvate, 10 mM l-ascorbate-2-phosphate (Sigma-Aldrich, Inc., St. Louis, MO), L-glutamine and non-essential amino acids. The induced cells were passaged using Accutase^@^ (Stem Cell Technologies, Vancouver, Canada) after 2 weeks of induction and defined as passage 1 (P1) iPSC-MSCs. DMEM supplemented with 10% FCS (Gibco), 1% penicillin-streptomycin (Gibco), 100ng ng/ml EGF (PeproTech) and 11ng ng/ml β-FGF (PeproTech) was used cultured the induced cells and was changed every 3 days. Passage 9 and passage 10 iPSC-MSCs were used in this study. Typical MSC characteristics such as expression of common MSC surface markers, self-renewal and multipotent differentiation potential were validated before experimentation. The human BM-MSCs used in this study were commercially purchased from Cyagen (Guangzhou, China).

**Flow cytometry analysis**

PBMCs were collected and stained with APC-eFluor 780 conjugated anti-CD3 (UCHT1), FITC conjugated anti-CD4 (RPA-T4), PerCP-Cy5.5 conjugated anti-CD8 (RPA-T8), APC conjugated anti-CD25 (BC96), Pe/Cy7 conjugated anti-CD69 (FN50), and PE conjugated anti-Foxp3 (PCH101) monoclonal antibodies (eBioscience, CA, USA). Generally, the CD69^+^ cells, CD4^+^CD69^+^ cells, CD8^+^CD69^+^ cells, CD4^+^CD25^+^CD69^+^ cells, CD4^+^Foxp3^+^ cells and CD4^+^CD69^+^Foxp3^+^ cells in lymphocytes or CD4^+^, CD8^+^ T cells were gated for analysis. iPSC-MSCs were harvested and stained with APC-H7 conjugated anti-human HLA-DR (G46-6, BD Biosciences, NJ, USA) and FITC conjugated anti-human HLA-ABC (W6/32, eBioscience, CA, USA) monoclonal antibodies, then the HLA expression profiles of iPSC-MSCs were analyzed by flow cytometry. A minimum of 80,000 events was collected in each analysis. Isotype-matched immunoglobulin was used as negative control for each assay.

**Quantitative real-time PCR**

*Foxp3* (forward primer: 5’-CAC CTG GCT GGG AAA ATG G-3’, reverse primer: 5’-GGA GCC CTT GTC GGA TGA T-3’), *IL-4* (forward primer: 5’-GCA CCG AGT TGA CCG TAA CA-3’, reverse primer: 5’-GCG AGT GTC CTT CTC ATG GT-3’), *IL-5* (forward primer: 5’-AGA GAC CTT GGC ACT GCT TT-3’, reverse primer: 5’-TTT CCA CAG TAC CCC CTT GC-3’) and *IL-13* (forward primer: 5’- TCT GCA ATG GCA GCA TGG TA-3’, reverse primer: 5’- GCA TCC TCT GGG TCT TCT CG-3’) expression levels in PBMCs co-cultured with iPSC-MSCs and BM-MSCs were measured using qPCR. Briefly, the total RNA of purified PBMCs were extracted using TRIzol Reagent (Invitrogen, CA, USA). cDNA of the samples was prepared using the synthesis kits from Takara (Japan). qPCR was performed using SYBR^®^ Green master mix (Applied Biosystems, CA, USA). The doublets of the reaction mixture were cycled once at 95ºC (5 sec) and 60ºC (1 min); 40 cycles at 95ºC (5 sec), 55ºC (30 sec), and 72ºC (30 sec); and a final cycle at 95ºC (5 sec), 60ºC (30 sec). *GAPDH* was applied as the normalized control, and the fold change was calculated as 2^-ΔΔCt^.

**Knockdown of IKKβ in iPSC-MSCs with short hairpin RNA**

Control lentiviral pLKO.1 vector and pLKO.1 vectors containing shRNAs for human IKKβ (RHS4533-NM_001556) were obtained from Open Biosystems. A total of three IKKβ knockdown iPSC-MSCs were constructed (shIKKβ-1: AAACTTAAAGCTGGTTCATATCT; shIKKβ-2: AAACAGGTGAGCAGATTGCCATC; shIKKβ-3: AAATTATTGACCTAGGATATGCC). To establish stable IKKβ knockdown iPSC-MSCs, iPSC-MSCs were infected twice with lentiviral particles for 18-20 hours at each time. Stably transduced cells were selected with puromycin (2 µg/ml). IKKβ knockdown in iPSC-MSCs was confirmed by qPCR, the primers used for IKKβ detection were forward–TTG TAG CAA GGT CCG TGG TC and reverse-GGG TGC AGA GGT TAT GTG CT. The PBMCs co-cultured with the transduced iPSC-MSCs were collected for flow cytometry analysis to validate the role of NF-κB in iPSC-MSC-mediated immunomodulation.

**References**

Bousquet, J., Khaltaev, N., Cruz, A.A., Denburg, J., Fokkens, W.J., Togias, A., Zuberbier, T., Baena-Cagnani, C.E., Canonica, G.W., van Weel, C.*, et al.* (2008). Allergic Rhinitis and its Impact on Asthma (ARIA) 2008 update (in collaboration with the World Health Organization, GA(2)LEN and AllerGen). Allergy *63 Suppl 86*, 8-160.

Xue, Y., Cai, X., Wang, L., Liao, B., Zhang, H., Shan, Y., Chen, Q., Zhou, T., Li, X., and Hou, J. (2013). Generating a non-integrating human induced pluripotent stem cell bank from urine-derived cells. PLoS One *8*.

# Additional tables and figures

## Additional table

**Table S1 High-resolution HLA typing for HLA-A, -B, -C, -DRB1 and -DQB1 of the MSCs and PBMCs utilized in this study.**

| Sample type | | HLA-A | | HLA-B | | HLA-Cw | | HLA-DRB1 | | HLA-DQB1 | |
| --- | --- | --- | --- | --- | --- | --- | --- | --- | --- | --- | --- |
| iPSC-MSCs | | 02:01 | 02:07 | 45:01 | 57:03 | 06:02 | 07:01 | 07:01 | 15:01 | 06:02 | 02:02 |
| BM-MSCs | | 01:01 | 33:03 | 40:01 | 58:01 | 03:02 | 03:04 | 01:01 | 03:01 | 02:01 | 05:01 |
| PBMCs | No. 1 | 02:01 | 30:01 | 13:02 | 15:11 | 03:03 | 06:02 | 07:01 | 09:01 | 02:02 | 03:03 |
|  | No. 2 | 02:03 | 24:20 | 38:02 | 55:02 | 01:06 | 07:02 | 09:01 | 14:54 | 05:02 | 03:03 |
|  | No. 3 | 02:07 | 33:03 | 46:01 | 58:01 | 03:02 | 01:02 | 09:01 | 03:01 | 02:01 | 03:03 |
|  | No. 4 | 11:01 | 24:02 | 51:02 | 55:02 | 12:03 | 15:02 | 09:01 | 15:01 | 06:01 | 03:03 |
|  | No. 5 | 01:01 | 33:03 | 37:01 | 51:01 | 06:02 | 07:02 | 04:07 | 10:01 | 05:01 | 03:01 |
|  | No. 6 | 02:01 | 24:02 | 35:01 | 40:01 | 03:03 | 03:04 | 04:01 | 15:01 | 06:02 | 03:01 |
|  | No. 7 | 02:03 | 11:01 | 27:04 | 40:01 | 04:03 | 12:02 | 12:02 | 16:02 | 05:02 | 03:01 |

## Additional figures


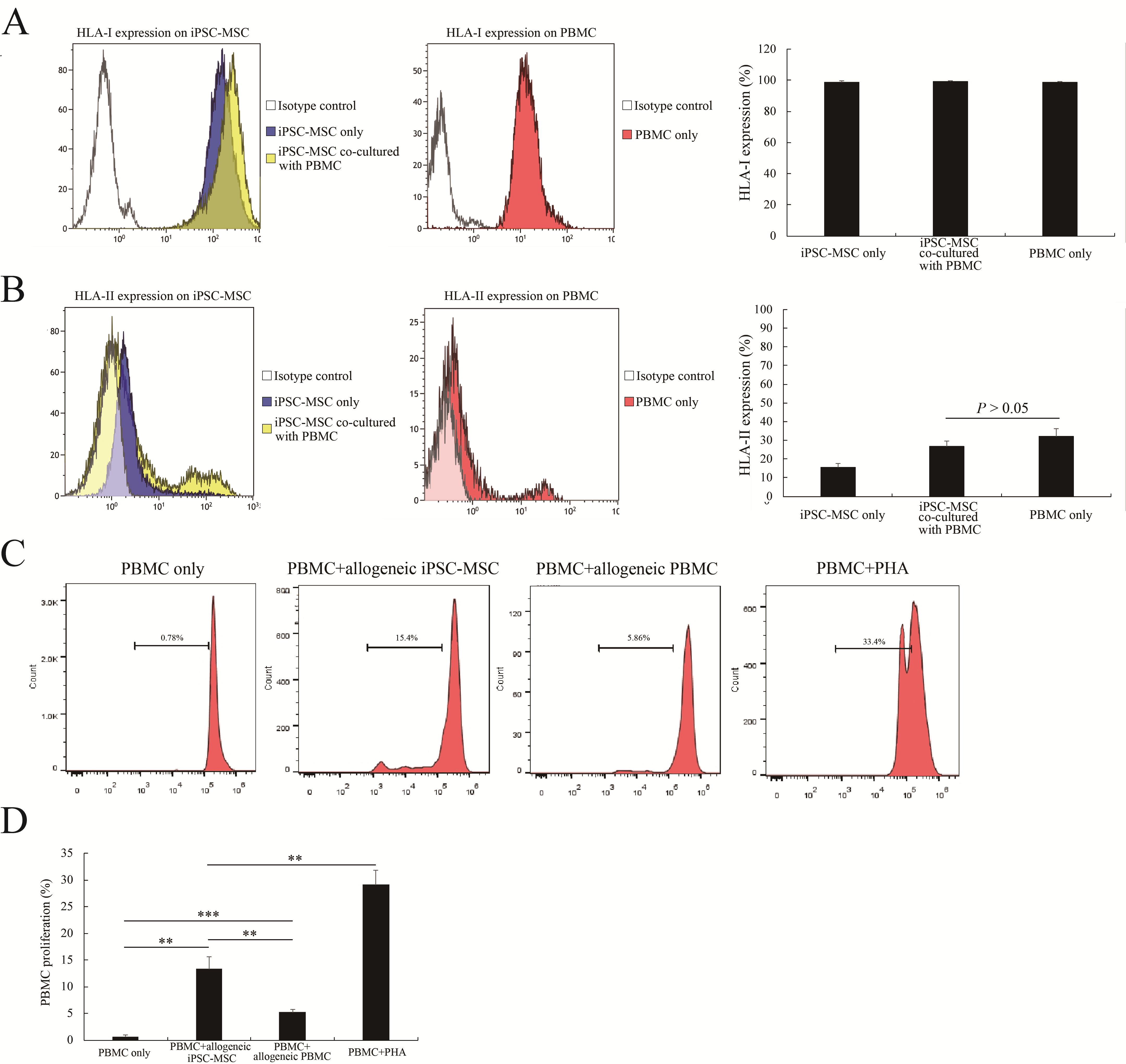


**Figure S1 The promotion effects of iPSC-MSCs on resting PBMCs was not only due to the allogeneic recognition.** A. Representative histogram and statistical analysis of HLA-I expression on iPSC-MSCs after co-culture with resting PBMCs as determined by flow cytometry. B. Representative histogram and statistical analysis of HLA-II expression on iPSC-MSCs after co-culture with resting PBMCs as determined by flow cytometry. C. Representative histogram of CFSE stained PBMC proliferation when co-cultured with allogeneic iPSC-MSCs/PBMCs. D. Statistical analysis of PBMC proliferation in C. (n = 6, **: *P* < 0.01, ***: *P* < 0.001 by a one-way analysis of variance and Dunnett T3 test for multiple comparisons for B, Kruskal–Wallis rank sum test followed by the Mann–Whitney U test for two-group comparisons for D)

**
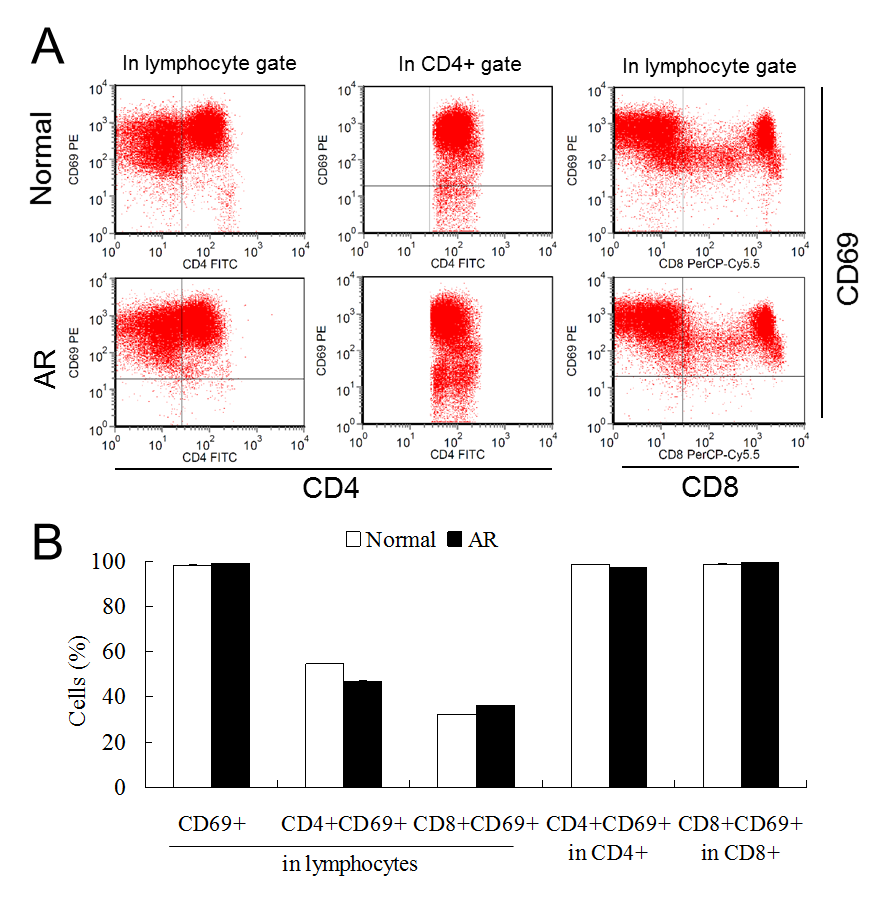
**

**Figure S2. The activation of CD4^+^, CD8^+^ T cells in both AR patients’ and normal PBMCs with the stimulation of PMA and ionomycin.** Flow cytometry analysis (A) and statistical analysis of CD69^+^ lymphocytes, CD69^+^CD4^+^ T cell in CD4^+^ T cells and CD69^+^CD8^+^ T cells in CD8^+^ T cells (B) in PBMCs. Abbreviations: AR, allergic rhinitis; PBMCs, peripheral blood mononuclear cells; PMA, phorbol 12-myristate 13-acetate.


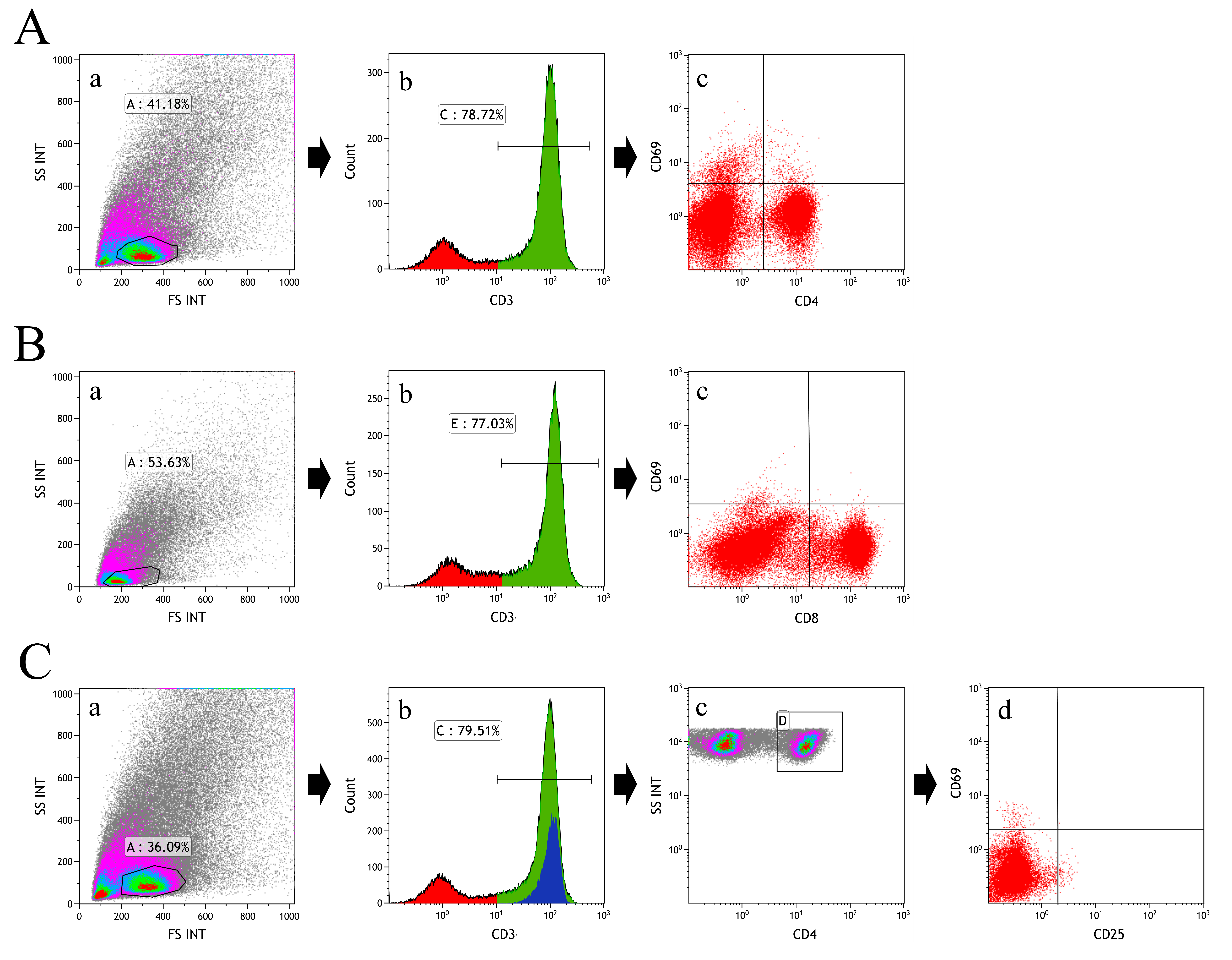


**Figure S3 Representative gating strategies for CD4^+^ and CD8^+^T cells in this study.** After co-cultured with stem cells, the CD3^+^CD4^+^CD69^+^ T cells (A), CD3^+^CD8^+^CD69^+^ T cells, (B) and CD3^+^CD4^+^CD25^+^CD69^+^ T cells (C) in PBMCs were gated using flow cytometry. (Ab Red: lymphocytes; Green: CD3^+^ T cells. Bb: Red: lymphocytes; Green: CD3^+^ T cells. Cb Red: lymphocytes; Green: CD3^+^ T cells; Blue: CD3^+^CD4^+^ T cells)


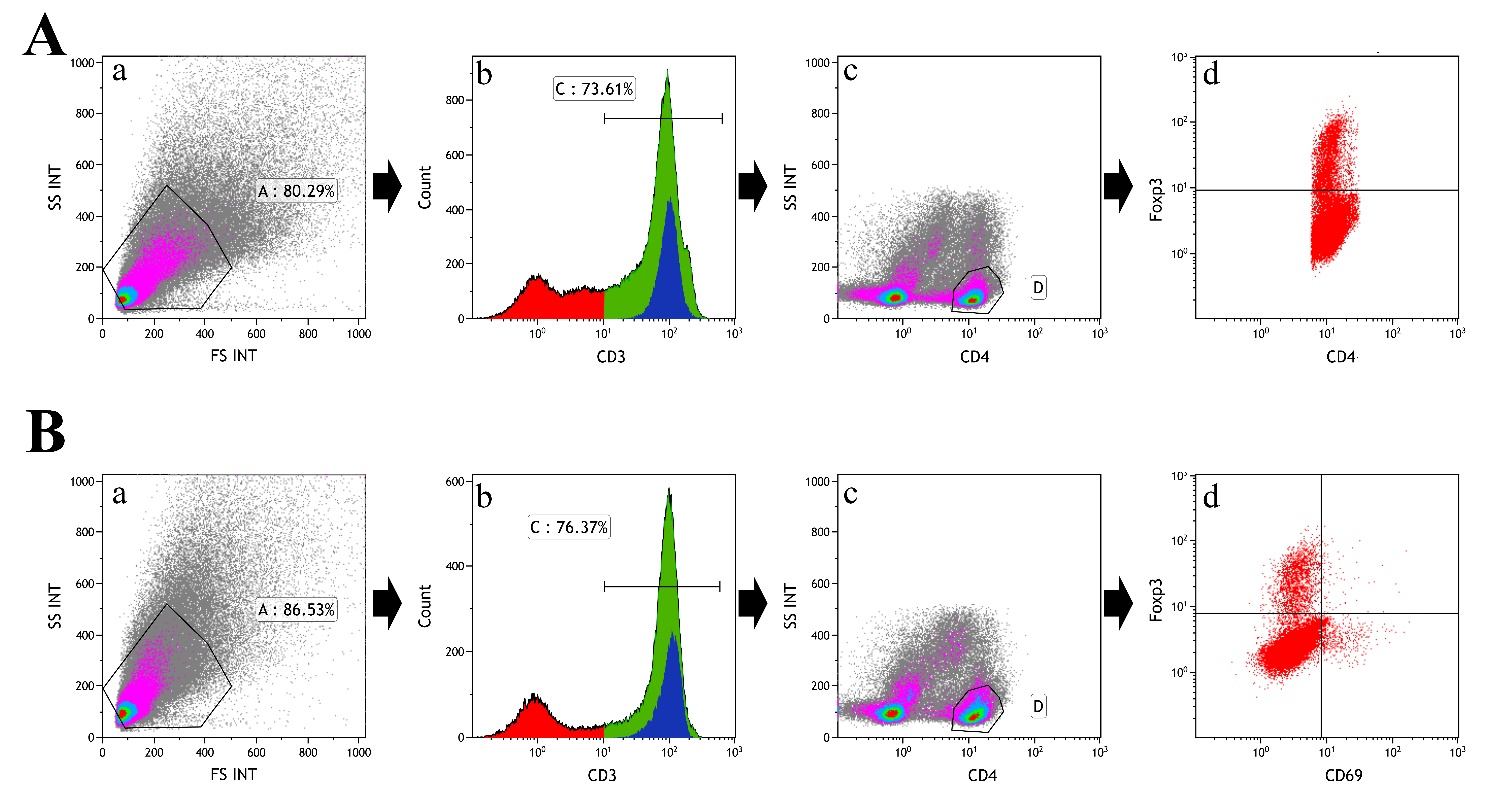


**Figure S4 Representative gating strategies for Treg cells in this study.** After co-cultured with stem cells, the CD3^+^CD4^+^Foxp3^+^ Treg cells (A) and CD3^+^CD4^+^CD69^+^Foxp3^+^ (B) in PBMCs from AR patients were gated using flow cytometry. (Ab Red: lymphocytes; Green: CD3^+^ T cells; Blue: CD3^+^CD4^+^ T cells. Bb: Red: lymphocytes; Green: CD3^+^ T cells; Blue: CD3^+^CD4^+^ T cells)

**Figure S5 Foxp3 mRNA expression in PBMCs from normal and AR patients after co-cultured with MSCs.** Statistical analysis of Foxp3 mRNA expression compared to control. (n = 3, *: *P* < 0.05, **: *P* < 0.01 by a one-way analysis of variance and Dunnett T3 test for multiple comparisons. Abbreviations: AR, allergic rhinitis; BM-MSC, bone marrow-derived mesenchymal stem cell; iPSC-MSCs, induced pluripotent stem cell-derived mesenchymal stem cells; PBMCs, peripheral blood mononuclear cells)


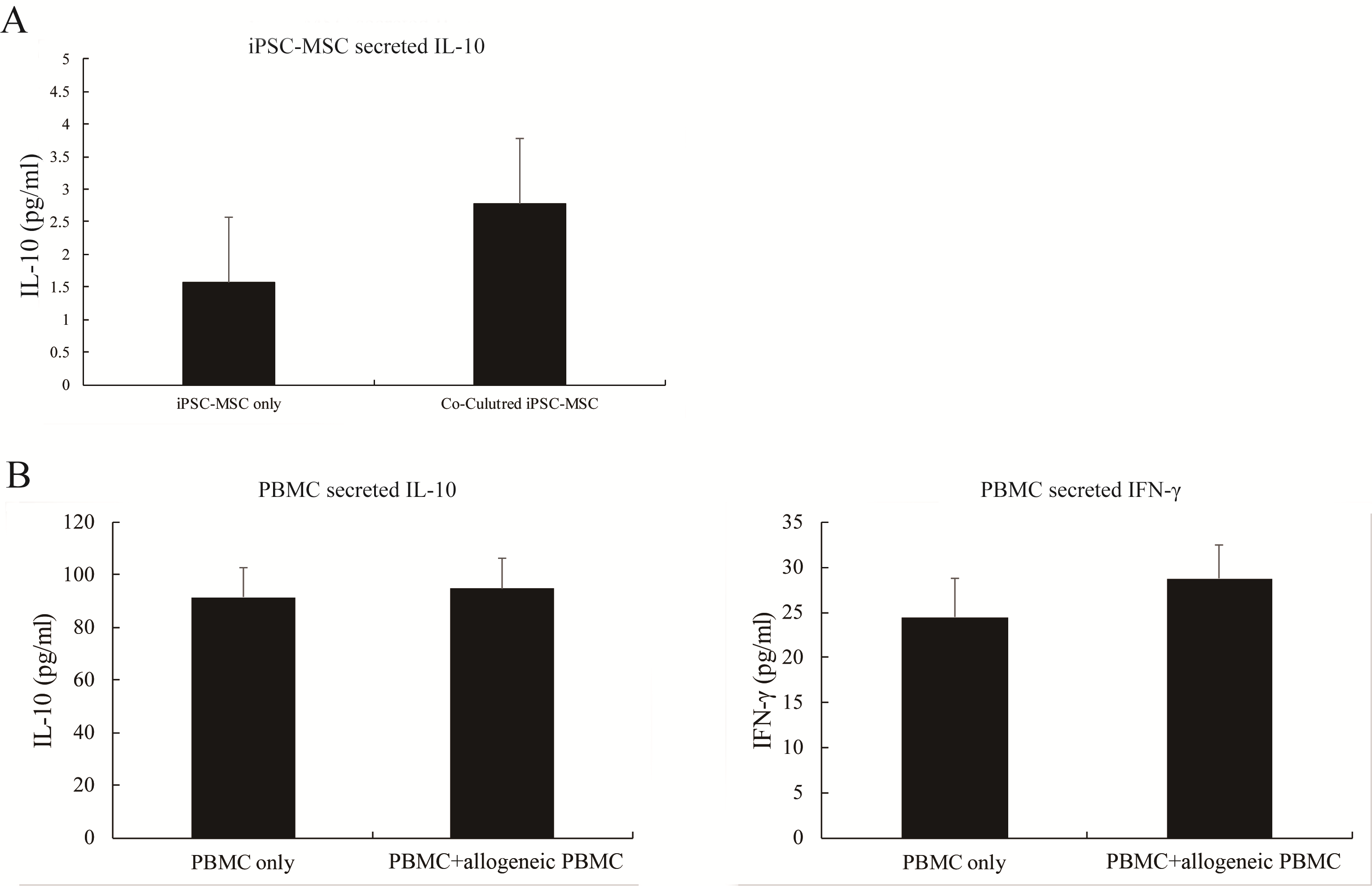


**Figure S6 IL-10 and IFN-γ levels secreted by the iPSC-MSCs/PBMCs under different conditions.** A. PBMCs were co-cultured with allogeneic iPSC-MSCs for 3 days, and iPSC-MSCs were cultured in the new medium for an additional 12 h after PBMCs were removed. Statistical analysis of IL-10 levels secreted by the separated iPSC-MSCs from the co-culture system as measured by ELISA. B. PBMCs were co-cultured with allogeneic PBMCs for 3 days. Statistical analysis of IL-10 and IFN-γ levels secreted by PBMCs when co-cultured with allogeneic PBMCs. (n = 6. Abbreviations: iPSC-MSCs, induced pluripotent stem cell-derived mesenchymal stem cells; PBMCs, peripheral blood mononuclear cells)

**Figure S7 PGE2 levels in PBMCs from healthy control and AR patients when co-cultured with MSCs and NS398.** Statistical analysis of PGE2 secretion level. (n = 3, *: *P* < 0.05, **: *P* < 0.01, ***: *P* < 0.001 by Kruskal–Wallis rank sum test followed by the Mann–Whitney U test for two-group comparisons. Abbreviations: AR, allergic rhinitis; BM-MSC, bone marrow-derived mesenchymal stem cell; iPSC-MSCs, induced pluripotent stem cell-derived mesenchymal stem cells; NS398, COX-2 inhibitor; PBMCs, peripheral blood mononuclear cells; PGE2, prostaglandin E2).


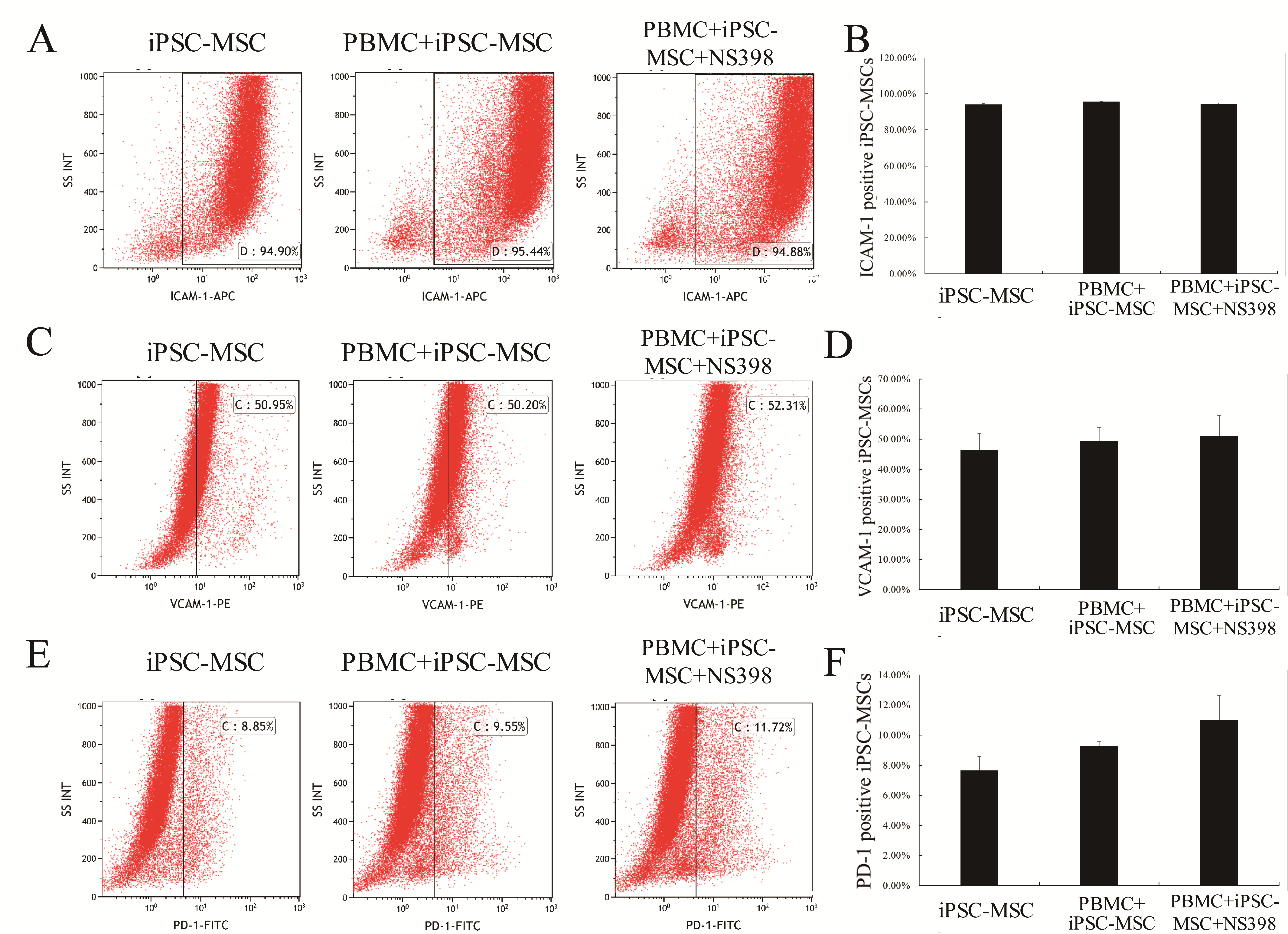


**Figure S8 ICAM-1, VCAM-1 and PD-1 expressions on iPSC-MSCs when co-cultured with PBMCs.** A. Representative flow cytometry cytograms of ICAM-1 expression on iPSC-MSCs when co-cultured with normal PBMCs; B. Statistical analysis of ICAM-1 on iPSC-MSCs under different conditions (n = 3); C. Representative flow cytometry cytograms of VCAM-1 expression on iPSC-MSCs when co-cultured with normal PBMCs; D. Statistical analysis of VCAM-1 on iPSC-MSCs under different conditions (n = 3); E. Representative flow cytometry cytograms of PD-1 expression on iPSC-MSCs when co-cultured with normal PBMCs; B. Statistical analysis of PD-1 on iPSC-MSCs under different conditions (n = 3). (Abbreviations: ICAM-1: intercellular adhesion molecule 1; iPSC-MSCs, induced pluripotent stem cell-derived mesenchymal stem cells; NS398, COX-2 inhibitor; PBMCs, peripheral blood mononuclear cells; PD-1: programmed cell death protein 1; VCAM-1: Vascular cell adhesion protein 1).
